# The In Vitro and In Vivo Anticancer Properties of Chalcone Flavokawain B through Induction of ROS-Mediated Apoptotic and Autophagic Cell Death in Human Melanoma Cells

**DOI:** 10.3390/cancers12102936

**Published:** 2020-10-12

**Authors:** You-Cheng Hseu, Yu-Chi Chiang, Yugandhar Vudhya Gowrisankar, Kai-Yuan Lin, Sheng-Teng Huang, Sirjana Shrestha, Geng-Ruei Chang, Hsin-Ling Yang

**Affiliations:** 1 Department of Cosmeceutics, College of Pharmacy, China Medical University, Taichung 40402, Taiwan; ychseu@mail.cmu.edu.tw (Y.-C.H.); dr.vgyugandhar@mail.cmu.edu.tw (Y.V.G.); 2 Department of Health and Nutrition Biotechnology, Asia University, Taichung 41354, Taiwan; 3 Chinese Medicine Research Center, China Medical University, Taichung 40402, Taiwan; 4 Research Center of Chinese Herbal Medicine, China Medical University, Taichung 40402, Taiwan; 5 Institute of Nutrition, College of Biopharmaceutical and Food Sciences, China Medical University, Taichung 40402, Taiwan; u101008303@cmu.edu.tw (Y.-C.C.); sirju10@mail.cmu.edu.tw (S.S.); 6 Department of Medical Research, Chi Mei Medical Center, Tainan 71004, Taiwan; d970712@mail.chimei.org.tw; 7 Department of Biotechnology, Chia Nan University of Pharmacy and Science, Tainan 71004, Taiwan; 8 School of Chinese Medicine, China Medical University, Taichung 40402, Taiwan; sthuang@mail.cmu.edu.tw; 9 Department of Veterinary Medicine, National Chiayi University, Chiayi 60054, Taiwan

**Keywords:** flavokawain B, melanoma cells, apoptosis, autophagy, ROS

## Abstract

**Simple Summary:**

Melanoma is the most dangerous type of skin cancer that develops from the pigment-producing cells known as melanocytes. One of the primary causes of melanoma is ultraviolet light (UV) exposure in those with low levels of the skin pigment melanin. Flavokawain B (FKB) is a naturally occurring chalcone, which is known to possess anti-proliferative pharmacological activity on various cancer cells. However, the effect of FKB on the anti-melanoma pharmacological role has not been investigated. Therefore, in this study, we explored the anti-melanoma properties of FKB on human melanoma cells and the cell death mechanisms that were mediated through the induction of reactive oxygen species (ROS) were investigated via in vitro and in vivo approaches. Our study results support promising application prospects of FKB in the treatment of human melanoma cancer.

**Abstract:**

Melanoma is the most prevalent type of skin cancer with high mortality rates. This study demonstrates the in vitro and in vivo anticancer properties of chalcone flavokawain B (FKB) induced ROS-mediated apoptosis and autophagy in human melanoma (human epithelial melanoma cell line A375 and/or human skin lymph node derived melanoma cell line A2058) cells. Cell viability was calculated by 3-(4,5-dimethylthiazol-2-yl)-2,5-diphenyltetrazolium bromide (MTT) assay and the expression patterns of various apoptosis, autophagy-associated proteins were determined by Western blot methods. Annexin V was detected by flow cytometry, whereas acidic vesicular organelles (AVOs) and intracellular ROS levels were measured by fluorescence microscopy. The in vivo anticancer properties of FKB were evaluated by xenografting the A375 cells into nude mice. The results convey that FKB inhibited cell viability, B-Raf proto-oncogene, serine/threonine kinase (BRAF)/extracellular signal-regulated kinase (ERK) expression in human melanoma cells. Caspase-3 activation, poly (ADP-ribose) polymerase (PARP) cleavage pathway, and Bcl2 associated X (Bax)/B-cell lymphoma 2 (Bcl-2) dysregulation were involved in the execution of apoptosis. Moreover, FKB-induced autophagy was observed through increased microtubule-associated protein 1A/1B-light chain 3B (LC3-II) accumulation and AVOs formation, which was also associated with an increase in sequestosome 1 (SQSTM1/p62), decreased protein kinase B (AKT)/mammalian target of rapamycin (mTOR) expressions, and dysregulated Beclin-1/Bcl-2 levels. Autophagy inhibitors [3-methyladenine (3-MA)/chloroquine (CQ)] and LC3 silencing suppressed FKB-induced apoptosis by decreasing caspase-3 in melanoma cells. The antioxidant *N*-acetylcysteine (NAC) diminished FKB-induced apoptotic and autophagic cell death. However, the inhibition of apoptosis decreased FKB-induced autophagy (LC3-I/II). The in vivo study confirmed that FKB inhibited melanoma growth in A375-xenografted nude mice. This study concluded that FKB is critically associated with the execution and generation of ROS-modulated apoptotic and autophagic cell death of melanoma cells. FKB also repressed tumor growth in xenografted nude mice. Therefore, flavokawain B might be a potential anti-tumor agent in human melanoma treatment.

## 1. Introduction

Every year, a significant number of newly identified skin cancers occur worldwide, and the cases of melanoma are rising higher than most other cancers [1]. Ultraviolet light (UV) radiation exposure is a major environmental risk factor for the growth of primary cutaneous melanoma. Elmwood and Gallagher identified that individuals with pale, un-acclimated white skin have a higher incidence of melanoma occurrence [2]. A total of 80% of deaths are due to malignant melanoma when compared with other nonmelanoma cancers [3] and occurring more commonly in man than in women [4]. Despite significant innovation in terms of the detection and treatment of melanoma, its prognosis remains poorly understood [5].

Cell death is an innate ability of cells and is controlled by different molecular pathways and mechanisms such as apoptosis, autophagy, and necroptosis. Molecular networks are important to the aforementioned processes and are linked to maintain a balance. Treatment strategy for cancer relies on anticancer therapy to cause tumor-specific cell death without destroying normal neighboring cells [6]. Autophagy and apoptosis are cellular mechanisms that regulate organelles and protein turnover in cells, and of cells inside organisms, respectively. Dialogue between autophagy and apoptosis occurs within the same cell or between different cells and takes on a key role in cancer, aging etc. [7].

Since the early 1990s, research on “apoptosis” or “programmed cell death” has increased tremendously because of its importance in biological phenomenon. During apoptosis, several morphological and biochemical changes occur such as blebbing, cell shrinkage, nuclear fragmentation, and chromatin condensation, which differs from other cell death pathways. It includes the activation of the caspase cascade. This process either begins either through the mitochondrial i.e., intrinsic, or death receptor mediated i.e., extrinsic, pathways. In mitochondrial pathway of apoptosis, cytochrome c is released into the cytosol, which binds to apoptotic protease activating factor-1 (Apaf-1) and activates procaspase-9 and then procaspase-3. On the other hand, death receptors such as Fas/CD95, DR3, and TNFR1 are involved in death receptor mediated pathway of apoptosis, which triggers caspase activation (caspase-8 and caspase-10), initiating a cascade of apoptotic events. However, activation of caspases is necessary for both pathways because it cleaves various key proteins [8,9]. 

Autophagy is a self-catabolic process that can be both good and bad. It maintains intracellular homeostasis and intricately entangles various facets of human health and diseases, including cancer [10,11]. Under stressful conditions, the lysosomal recycling of amino acids takes place, which prolongs cell survival. Several molecular and cell signaling pathways regulate autophagy. Microtubule-associated light chain 3 (LC3) is a major protein in autophagy pathway that functions as autophagy substrate selector and is involved in autophagosome biogenesis. LC3 is a commonly used marker for autophagosomes [12]. Moreover, a key mechanism that controls autophagy has to modulate the interaction between Beclin-1 and the anti-apoptotic members of Bcl-2 family (e.g., Bcl-2, Bcl-X_L_, and Mcl-1). Various proteins and compounds regulate this binding that can either promote or suppress the Bcl-2/Beclin-1 interaction ultimately leading to the repression or activation of autophagy, respectively. Additionally, in normal cells, a master regulator called the mammalian target of rapamycin (mTOR) kinase regulates autophagy and upstream signaling pathways [13]. The availability of nutrient or growth factor triggers the PI3K/AKT/mTOR pathway thus leading to the inhibition of autophagy and stimulating cell growth and proliferation. However, the limitation of nutrient or growth factors, hypoxia, and other stresses neutralize the pathway and leads to the induction of autophagy [14].

Autophagy acts as tumor suppressor as well as protector of cancer cell survival [15]. Cellular homeostasis is maintained by basal autophagy, which is characterized by the removal of protein aggregates and damaged organelles. Conversely, starvation-induced autophagy can extend the survival of cells by reusing amino acid and energy, which are crucial to keep cells fit and preserve viability [16]. High metabolic stressors are more common in cancer cells than in normal cells due to tumors being more dependent on autophagy for survival, suggesting that therapeutics for favorable cancer cell targeting is possible from pharmacological modulation of autophagy. By explaining the particular role for autophagy at different stages of cancer as well as determining cell type and genetic context dependence shows a way forward in developing new cancer therapies and prevention strategies. 

ROS is derived from the metabolism of oxygen and suggested to be a critical mediator in apoptosis and autophagy-mediated cell death. ROS is also necessary for the maintenance of physiological homeostasis [17,18]. Earlier studies have shown that activation of apoptosis due to ROS is a result of losses in mitochondrial membrane potential, release of cytochrome c, caspase-9, -3 activation, cleavage of PARP, downregulation of Bcl-2, and upregulation of Bax, pro-apoptotic protein, in various cell types. Evidence has suggested that there is participation of ROS in controlling several autophagy regulating proteins, such as mTOR in cancer inhibition [19]. Moreover, other researchers have indicated that chemotherapeutic agents can induce cell death through the enhancement of ROS generation [20,21]. 

More than half a century after the introduction of chemotherapy for cancer treatment, various compounds emerged from natural phytochemicals or as drugs produced from natural products [22]. Extracts derived from roots of kava consumed in the pacific islands are correlated with lower prevalence of cancer [23]. *Piper methysticum*, commonly known as kava-kava, is a perennial shrub of the pacific islands. Kava extracts are classified as follows: chalcone and kavalactone. Chalcones act as a precursor for flavonoids and showed antinociceptive, anti-inflammatory, and anti-cancer effects [24]. Three different types of naturally occurring chalcones include flavokawain A, B, and C [25]. Recently, the safety of kava use and its association with liver maladies has been questioned. It has been reported that its overdose, co-medication, and poor-quality raw materials could all lead to hepatotoxicity [26]. In contrast, herbal–drug interaction can also account for the rare hepatotoxicity related with its use [27]. In 2014, Germany’s Federal Administrative Court also ended a ban on kava being consumed [28]. Flavokawain B (FKB) activated apoptosis and showed anticancer properties in vitro and in vivo against several cancers [29,30,31]. For example, FKB treatment increased intracellular ROS concentration and decreased mitochondrial membrane potential (MMP) leading to inhibition of A549 (human non-small cell lung carcinoma cell line) cell viability [32]. In another study, FKB had shown its important role in the execution and propagation of ROS-mediated apoptotic and autophagic cell death of lung adenocarcinoma cells [33]. Additionally, FKB induced protective autophagy in human glioblastoma multiforme (GBM) cells, suggesting that the combination treatment of FKB with autophagy inhibitors may potentially be an effective therapeutic strategy for GBM [34]. In one of our recent studies, FKB and doxorubicin showed the synergistic anti-tumor effects in gastric cancer cells indicating that it might be a promising therapeutic approach [35]. However, as far as we know, this is the first study to demonstrate molecular aspects of FKB induced ROS-mediated apoptosis and autophagic cell death mechanisms both in vitro and in vivo using human melanoma (human epithelial melanoma cell line A375 and/or human skin lymph node derived melanoma cell line A2058) cells. 

## 2. Results

### 2.1. FKB Suppressed Human Melanoma Cell Growth

**Figure 1:**
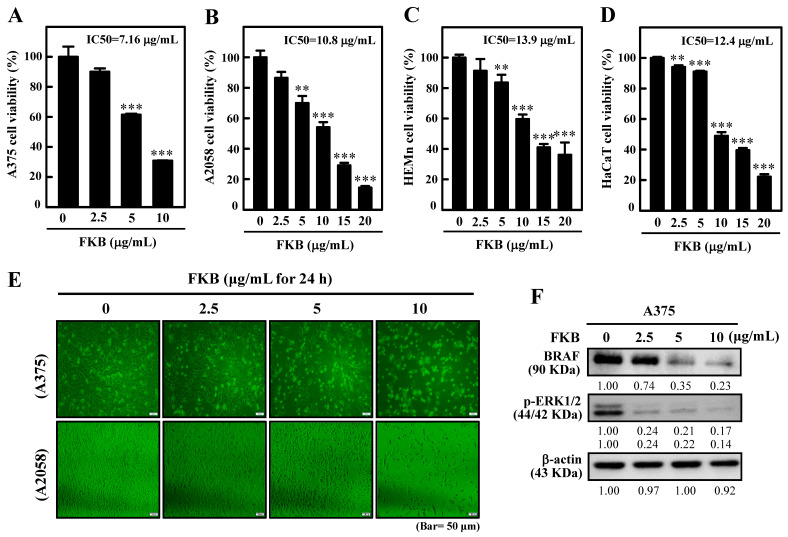
Flavokawain B (FKB) inhibited the growth of human melanoma cells. (**A**–**D**) human epithelial melanoma cell line A375, human skin lymph node derived melanoma cell line A2058, human primary epidermal melanocyte cell line (HEMn), and human skin keratinocyte cell line (HaCaT) cells were exposed to 0–20 µg/mL concentrations of FKB for 24 h. Then, 3-(4,5-dimethylthiazol-2-yl)-2,5-diphenyltetrazolium bromide (MTT) assay was carried out to determine cell viability. The IC_50_ value for each cell type was determined as described in the methods section (inset: IC_50_ values of respective cell lines). (**E**) FKB mediated morphological changes in A375 and A2058 cells were examined using the phase-contrast microscope. (**F**) Expression patterns of BRAF and p-ERK proteins were determined by Western blot to discover the effect of FKB concentrations (0–10 μg/mL) for 24 h. β-actin was used as an internal control. Each value was expressed as mean ± standard deviation of three experiments. Statistical significance was assigned as ** *p* < 0.01 and *** *p* < 0.001 as compared to the untreated control cells.

We first tested the FKB’s anti-cell proliferation efficacy in A375, A2058, human primary epidermal melanocyte cell line (HEMn), and human skin keratinocyte cell line (HaCaT) cell lines through their respective half maximal inhibitory concentration (IC_50_) values. Different concentrations of FKB were exposed for all cell lines for the indicated time (0–20 µg/mL, 24 h) and their IC_50_ values were determined. Furthermore, 3-(4,5-dimethylthiazol-2-yl)-2,5-diphenyltetrazolium bromide (MTT) data showed that FKB treatment significantly reduced A375 and A2058 cell survival in a dose-dependent manner with IC_50_ values of 7.6 and 10.8 μg/mL, respectively, indicating the susceptibility of these cancer cells to FKB treatment. Interestingly, FKB has been found to show less cytotoxicity in normal HEMn and HaCaT cells with corresponding IC_50_ values of 13.9 and 12.4 μg/mL, respectively (Figure 1A–D). Moreover, it was also found that FKB (0–10 µg/mL, 24 h) induced abrupt morphological changes (shrinkage of cells) in A375 and A2058 cells signifying the anti-tumor properties of FKB through the inhibition of cell growth and survival (Figure 1E). When compared to untreated control cells, Western blot data showed that FKB treatment (0–10 µg/mL, 24 h) dose-dependently suppressed the expressions of BRAF and its downstream p-ERK1/2 proteins in BRAF-expressing melanoma A375 cells (Figure 1F).

### 2.2. FKB Induced Apoptosis in Human Melanoma Cells

**Figure 2:**
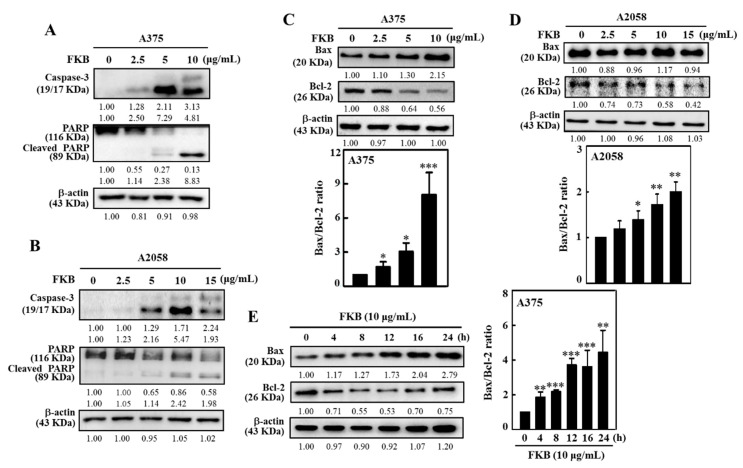
FKB induced apoptosis in human melanoma cells. Different concentrations of FKB were treated to A375 (0–10 μg/mL) and A2058 (0–15 μg/mL) cells for 24 h. (**A**,**B)** The expression levels of FKB-induced activation of caspase-3 and PARP cleavage were measured by Western blot in both A375 and A2058 cells. (**C**,**D**) The expression levels of apoptosis activator; Bax and inhibitor proteins; Bcl-2 were measured by Western blot in both A375 and A2058 cells. Data were expressed as fold-over control of the Bax/Bcl-2 ratio. **(E)** 10 μg/mL of FKB was treated to A375 for 0–24 h. The effect of time on the expressions of Bax and Bcl-2 proteins was measured by Western blot. β-actin was used as an internal control. The Bax/Bcl-2 ratio was represented as fold-over control whose value was arbitrarily assigned as one. Each value was expressed as mean ± standard deviation (SD) of three experiments and the statistical significance was assigned as * *p* < 0.05, ** *p* < 0.01, and *** *p* < 0.001 as compared to the untreated control cells.

We speculated that FKB might be playing a pivotal role in the activation of various proteins that were involved in the induction of apoptosis and/or autophagy in these cells because FKB exhibited cytotoxic effects in melanoma cells. Therefore, the effect of FKB with different concentrations (0–10 μg/mL) treated for 24 h was determined in A375 and A2058 cells. The expression patterns of caspase-3, PARP, Bax, and Bcl-2 proteins were determined by the Western blot. In comparison with untreated control cells, FKB dose-dependently activated the expression of caspase-3 in A375 and A2058 cells by causing the proteolytic cleavage of PARP (116KDa to 89 KDa fragment) (Figure 2A,B). PARP is an important protein characteristic of the apoptosis process [36]. Thus, it is suggested that FKB induced apoptosis in human melanoma A375 and A2058 cells via caspase-3 activation and PARP cleavage. 

The Bcl-2 family consists of most notable proteins that are involved in either promoting (Bax) or inhibiting (Bcl-2) thus activating mitochondrial outer membrane permeabilization (MOMP), which is crucial for the intrinsic pathway of apoptosis [37]. FKB treatment (0–10 μg/mL, 24 h) dose-dependently favored the expression of activator Bax with a dramatic decrease in the expression of inhibitor Bcl-2 in A375 and A2058 cells (Figure 2C,D) and signified the FKB-mediated apoptosis activation in melanoma cells. The quantified ratio of Bax/Bcl-2 was disrupted by FKB in a dose-dependent manner suggesting the induction of apoptosis (Figure 2C,D). Additionally, 10 μg/mL FKB treated A375 cells for 0–24 h showed that FKB mediated Bax and Bcl-2 expressions and their ratio also indicated a similar effect of favoring the activation of apoptosis (Figure 2E). 

### 2.3. FKB Induced Early and Late Apoptotic Cell Death in Melanoma Cells

**Figure 3:**
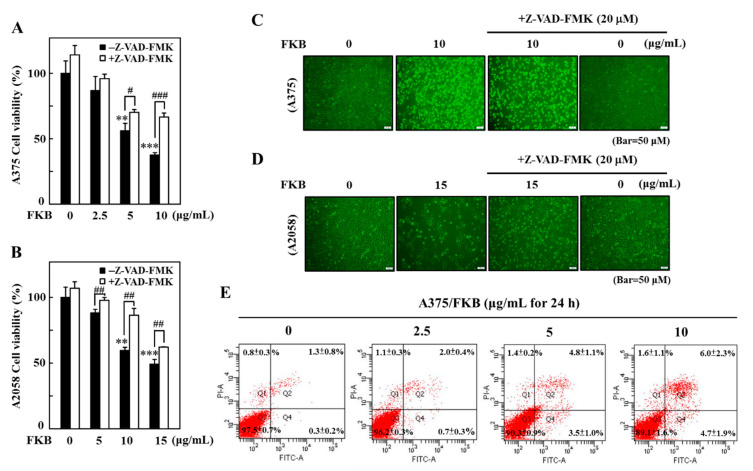
FKB induced apoptotic cell death of A375 and A2058 cells. Cells were pretreated with pan-caspase inhibitor z-Val-Ala-Asp fluoromethyl ketone (Z-VAD-FMK) (20 μM) for 1 h followed by treatment with various concentrations of FKB (0–10 μg/mL for A375 and 0–15 μg/mL for A2058) for 24 h. (**A**,**B**) MTT assay was performed to measure the viability of A375 and A2058 cells. (**C**,**D**) Changes in A375 and A2058 cell membrane morphology were examined under a phase-contrast microscope. (**E**) Flow cytometry analysis of FKB-mediated early and late apoptotic cell death in A375 cells using annexin V-fluorescein isothiocyanate (FITC) and propidium iodide (PI) staining. A375 cells were exposed to 0, 2.5, 5, and 10 μg/mL of FKB for 24 h. The results in each quadrant were interpreted as described in the methods section. Each value was expressed as mean ± standard deviation (SD) of three experiments. Statistical significance was assigned as ** *p* < 0.01 and *** *p* < 0.001 as compared to untreated control cells. ^#^ *p* < 0.05, ^##^ *p* < 0.01, and ^###^ *p* < 0.001 as compared to the treated cells.

Apoptotic caspase activation results in the activation or inactivation of different substrates, and a cascade of signaling events are evolved, which control degradation of cellular components [36]. Using the caspase inhibitor z-Val-Ala-Asp fluoromethyl ketone (Z-VAD-FMK), we tested the effect of FKB on the activation of apoptotic caspase signals as well as the viability and morphological aberrations in A375 and A2058 cells. Pretreatment of A375 and A2058 cells by 20 μM Z-VAD-FMK significantly downregulated the FKB-mediated cell death as revealed by the MTT data. Compared to the A2058 cells, this effect was more prominent in A375 cells at 10 μg/mL of FKB concentration (Figure 3A,B). Consistent with MTT data, the caspase inactivation using Z-VAD-FMK suppressed the alterations in membrane morphology that were caused by FKB in A375 and A2058 cells (Figure 3C,D). These data showed that there is a key role of caspases in cell death caused by apoptosis in melanoma cells induced by FKB. 

Annexin V, a cellular protein from annexin group, is used to detect apoptotic cells because it has an ability to bind phosphatidylserine, an apoptosis outer surface marker on the plasma membrane [38]. Double staining of annexin V with fluorescein isothiocyanate (FITC) and propidium iodide (PI) detected the FKB-induced apoptosis or necrosis in melanoma cells. Flow cytometry data revealed that 0–10 μg/mL of FKB treated for 24 h dose-dependently increased early (PI: negative/annexin V: positive) and late apoptosis (PI: positive/annexin V: positive cells) in A375 cells. The percentages of early and late apoptotic cells at different FKB concentrations are 0.3 ± 0.2% and 1.3 ± 0.8% (control), 0.7 ± 0.3% and 2.0 ± 0.4% (2.5 μg/mL), 3.5 ± 1.0% and 4.8 ± 1.1% (5 μg/mL), and 4.7 ± 1.9% and 6.0 ± 2.3% (10 μg/mL) (Figure 3E). These data suggested that FKB induced early and late apoptotic cell death in melanoma cells. 

### 2.4. FKB Increased LC3-II Accumulation and Activated Autophagy in Melanoma Cells 

**Figure 4:**
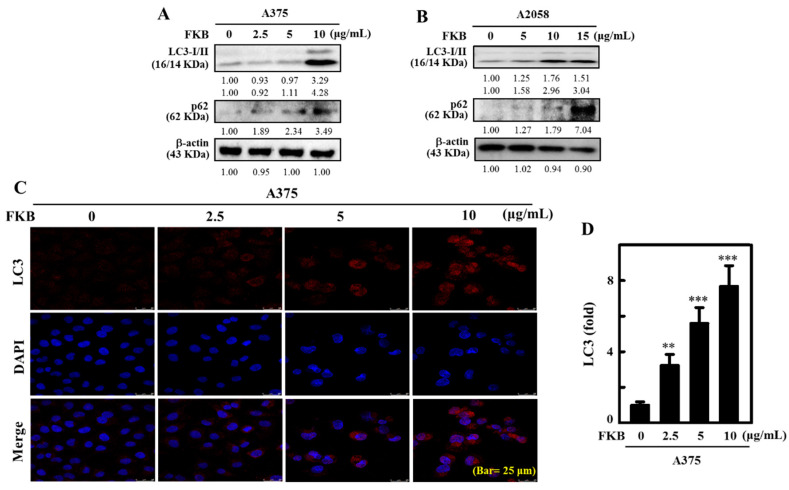
FKB induced autophagy LC3-II and sequestosome 1 (p62) expression in human melanoma cells. (**A**) A375 and (**B**) A2058 cells were treated with various concentrations of FKB (0–10 or 0–15 μg/mL) for 24 h. These cells were subjected to Western blot analysis to determine the conversion of LC3-I to LC3-II and expression of p62 proteins. (**C**,**D**) FKB (0–10 μg/mL) was treated to A375 cells for 24 h. Immunofluorescence staining (100× magnification) was used to measure the accumulation of LC3. The data were expressed as fold-over untreated control cells. Each value was expressed as mean ± standard deviation (SD) of three experiments. Statistical significance was assigned as ** *p* < 0.01, and *** *p* < 0.001 as compared to untreated control cells.

LC3-II is an autophagosomal marker that reflects lysosomal turnover and autophagy activity in the cells. The detection of LC3-II by Western blot and/or immunofluorescence are reliable methods for monitoring autophagy in cells [39]. p62, also known as sequestosome 1 (SQSTM1), is a classical multifunctional receptor protein and used as a marker protein to identify autophagic flux. It plays a role in the proteasomal degradation of ubiquitinated proteins and is located throughout the cell and in many signal transduction pathways. It attaches to LC3 at a specific site and then degrades itself when autophagy occurs [40]. The effect of FKB in activation of LC3-I/II in A375 and A2058 cells was tested. Western blot data showed that LC3-II and p62 protein expression increased as the concentrations of FKB increased from 0–10 μg/mL for A375 cells and from 0–15 μg/mL for A2058 cells (Figure 4A,B). LC3 accumulation in A375 cells was further determined using a fluorescence method. Consistent with the Western blot data, immunofluorescence images showed upregulation of LC3 occurred in dose-dependent manner in A375 cells (Figure 4C). This effect was statistically significant and measured as approximately eight-fold in FKB-treated cells in comparison to control cells (Figure 4D). These data provided strong evidence that FKB activated autophagy via LC3-II signaling cascades in melanoma cells. 

### 2.5. FKB Enhanced AVO Formation in A375 and A2058 Cells 

**Figure 5:**
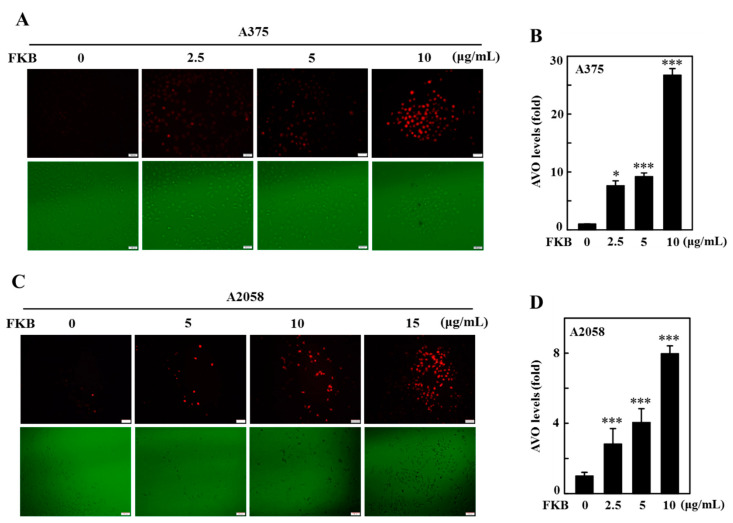
FKB triggered acidic vesicular organelle (AVO) formation in A375 and A2058 cells. A375 and A2058 cells were exposed to different concentrations of FKB (0–10 or 0–15 μg/mL) for 24 h. Acridine orange stain was used to detect the AVOs formation that can be observed through a red filter fluorescence microscope (100× magnification). (**A**,**B**) Compared to the untreated control cells, FKB treatment dose-dependently significantly upregulated the formation of AVOs in A375 and (**C**,**D**) A2058 cells. Histogram depicting the number of acridine orange (AO) stained cells. The data were expressed as fold-over untreated control cells in which control was assigned as one. Each value was expressed as mean ± standard deviation (SD) of three experiments. Statistical significance was assigned as * *p* < 0.05, and *** *p* < 0.001 as compared to untreated control cells.

Autophagy is characterized by the formation of acidic vesicular organelles(AVOs) and is represented by accumulation of LC3 levels [41]. Since FKB was able to induce the accumulation of LC3 in melanoma cells, using the fluorescence microscopy we further detected AVOs formation in these cells with acridine orange (AO) staining to confirm the role of FKB mediated autophagy. Similar to the LC3-II accumulation, FKB treatment (0–10 or 0–15 μg/mL) dose-dependently increased AVOs formation, indicated by red fluorescence, which in A375 cells was ~27-fold (Figure 5A,B) and A2058 cells was ~8-fold (Figure 5C,D), which leads to the induction of autophagy flux. 

### 2.6. FKB Dysregulated Beclin-1 and Bcl-2 Ratio and Reduced AKT Phosphorylation and mTOR Expressions Leading to Autophagy in Human Melanoma Cells

**Figure 6:**
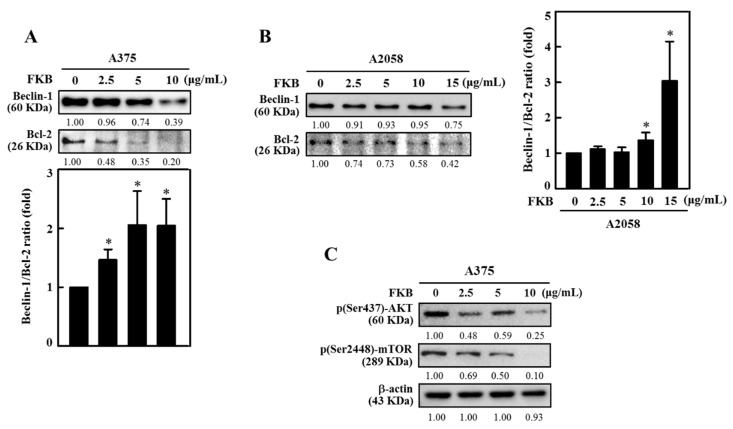
FKB dysregulated Beclin-1/Bcl-2 ratio and suppressed p-AKT/p-mTOR expressions in melanoma cells. Cells were treated with increasing concentrations of FKB (0–10 and 0–15 μg/mL) for 24 h. After the incubation period, cells were harvested and subjected to the Western blot method as described in the methods section. (**A**,**B**) FKB dose-dependently significantly upregulated the ratio between Beclin-1 and Bcl-2 in both A375 and A2058 cells. (**C**) FKB dose-dependently suppressed the phosphorylation of AKT and mTOR proteins. Both these effects shifted the cellular homeostasis towards autophagy. Each value was expressed as mean ± standard deviation (SD) of three experiments. Statistical significance was assigned as * *p* < 0.05 compared to untreated control.

Beclin-1 is necessary to initiate autophagy and associated together with Vps34, and Vps15 to form a core complex of Beclin1-Vps34-Vps15. Additionally, Beclin-1 is an important determining factor for cells that undergo autophagy or apoptosis. Bcl-2 binds to Beclin-1 and reduces its pro-autophagic function, but Beclin-1 is unable to neutralize the anti-apoptotic function of Bcl-2 [10,42]. We explored the effects of FKB concentration on homeostasis for Beclin-1 and Bcl-2 protein expressions. Western blot data indicated that FKB (0–10 μg/mL or 0–15 μg/mL for 24 h) dose-dependently reduced the expressions of Beclin-1 and Bcl-2 proteins in both A375 and A2058 cells (Figure 6A,B). Notably, FKB dysregulated the ratio between Beclin-1 and Bcl-2 proteins that leads to shifting the cellular fate to autophagy (low Bcl-2 expression) in both A375 and A2058 cells and this effect was statistically significant (Figure 6A,B). 

The AKT/mTOR pathway is very important pathway that regulates the cell cycle and is involved in cell proliferation, cellular quiescence, and many forms of cancers. AKT/mTOR is one of the critical signaling pathways and negatively impacts autophagy function [43]. The effects of FKB on AKT/mTOR expression in A375 cells was demonstrated in this study. When compared to untreated control cells FKB dose-dependently (0–10 μg/mL, 24 h) downregulated phosphorylated AKT (Ser437) and mTOR (Ser2448) expression levels as shown by Western blot (Figure 6C). These data supported that FKB activated autophagy in A375 cells and inhibited the AKT/mTOR pathway. 

### 2.7. FKB Accelerated Autophagy in A375 Cells as a Death Mechanism 

**Figure 7:**
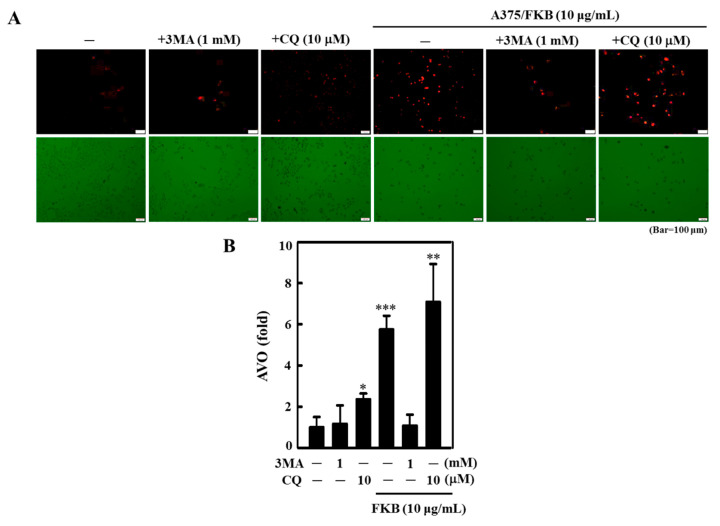
Effect of autophagy inhibitors on the FKB-mediated AVOs formation in A375 cells. (**A**,**B**) A375 cells were pretreated with 3-methyladenine (3-MA) (1 mM) or chloroquine (CQ) (10 μM) for 1 h followed by FKB treatment (10 μg/mL) for 24 h and then stained with acridine orange (AO) to observe the AVOs formed in the cells. These AVOs were visualized using the fluorescence microscope under a red filter (100× magnification). The fluorescence intensity was proportionate to the number of AVOs formed in the cells. Histogram representing the fold change of AVOs formation in different experimental conditions, in which the control value was arbitrarily assigned as one. Each value was expressed as mean ± standard deviation (SD) of three experiments. Statistical significance was assigned as * *p* < 0.05, ** *p* < 0.01, and *** *p* < 0.001 as compared to the untreated control cells.

To further evaluate the role of FKB-mediated autophagy in A375 cells, the pharmacological autophagy inhibitors 3-methyladenine (3-MA) and chloroquine (CQ) were used to disrupt the lysosomal function during the autophagy. Fluorescence results when compared to the FKB alone treatment showed that pretreatment with 3-MA (1 mM) downregulated LC3-II accumulation in early stages of autophagy and led to the prevention of FKB-induced (10 µg/mL) AVOs formation in A375 cells (Figure 7A,B). Conversely, CQ pretreatment (10 µM) showed a significant appearance of AVOs in A375 cells (Figure 7A,B), confirming that there was FKB-induced autophagy flux in A375 cells. 

**Figure 8:**
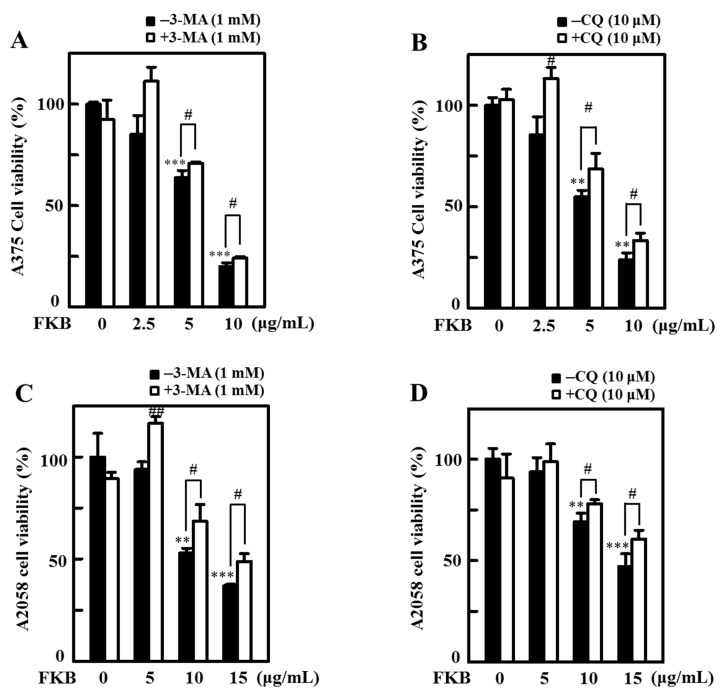
FKB induced autophagy signaling cascades mediated the cell death mechanisms in human melanoma cells. (**A**,**B**) A375 and (**C**,**D**) A2058 cells were pretreated with early and late autophagy inhibitors (1 mM 3-MA, and 10 μM CQ) for 1 h followed by FKB (0–10 or 0–15 μg/mL) treatment, respectively, for 24 h. MTT assay was performed to determine cell viability. Each value was expressed as mean ± standard deviation (SD) of three experiments. Statistical significance was assigned as ** *p* < 0.01 and *** *p* < 0.001 as compared to the untreated control cells. ^#^ *p* < 0.05 and ^##^ *p* < 0.01 as compared to the FKB alone treated cells.

The MTT cell viability data obtained from both A375 (Figure 8A,B) and A2058 (Figure 8C,D) cells suggest early (3-MA) and late (CQ) autophagy inhibitors significantly changed the FKB-induced cell death in these cell lines. These results elucidate FKB-triggered autophagy in melanoma cell death mechanism.

### 2.8. FKB Induced ROS Triggered Cell Death of Melanoma Cells 

**Figure 9:**
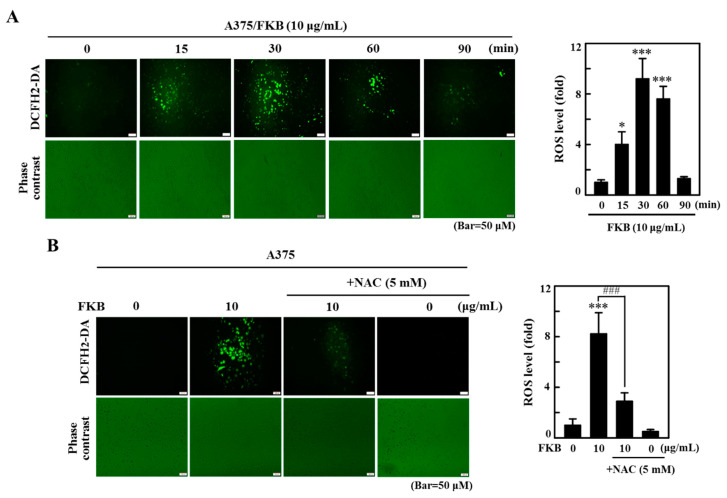
Intracellular ROS was induced by FKB in A375 cells. (**A**) A375 cells were treated with FKB (10 μg/mL) for 0–90 min. (**B**) Cells were pretreated with or without 5 mM *N*-acetylcysteine (NAC) (a ROS inhibitor) for 1 h followed by FKB (10 μg/mL) for 30 min. The non-fluorescent probe 2′-7′dichlorofluorescin diacetate (DCFH_2_-DA) was used to measure the intracellular ROS generation. DCFH_2_-DA reacted with cellular ROS and metabolized into fluorescent dichlorofluorescein (DCF), which was proportionate to the generation of ROS. The data were represented as fold-over change in the ROS levels compared to the control, which was arbitrarily assigned as one. Each value was expressed as mean ± standard deviation (SD) of three experiments. Statistical significance was assigned as * *p* < 0.05 and *** *p* < 0.001 compared to untreated control cells. ^###^ *p* < 0.001 compared to FKB alone treated cells.

ROS are critical mediators in apoptosis and autophagy. Excessive ROS eventually induce mitochondrial dysfunction leading to death of cell and play a significant role in the maintenance of physiological homeostasis [17,44]. To examine the role of ROS in the cell death mechanisms mediated by FKB, the effect of time on ROS production was demonstrated. A375 cells were incubated with FKB (10 µg/mL) at different time points (0–90 min) and the ROS produced intracellularly was measured with the dichlorofluorescein (DCF) fluorescence method. The data revealed that FKB increased the intracellular ROS production with a maximum production observed at 30 min (Figure 9A). However, cells pretreated with *N*-acetylcysteine (NAC) (5 mM, a ROS inhibitor) 30 min prior to FKB, significantly suppressed intracellular ROS levels (Figure 9B). The results show that FKB was involved in the induction of ROS production in A375 cells. 

**Figure 10:**
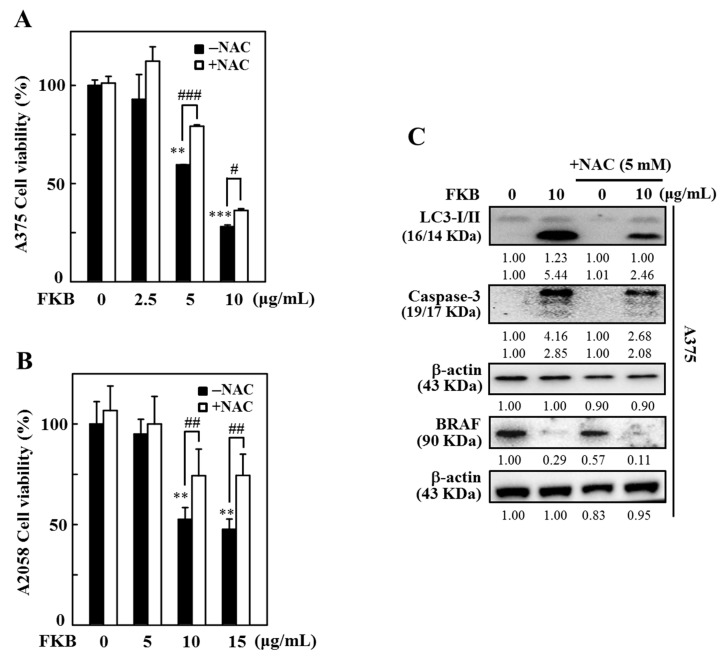
ROS-mediated apoptotic and autophagy cell death in A375 cells was induced due to FKB. A375 and A2058 cells were pretreated with 5 mM NAC for 1 h then followed by treatment with 0–10 and 0–15 μg/mL of FKB, respectively, for 24 h. (**A**,**B**) Cells were subjected to MTT assay to measure the percentage of cell viability. (**C**) Western blot was used to measure the expression levels of LC3-I/II, caspase-3, and BRAF proteins both in the absence or presence of NAC pretreatment conditions. β-actin acts as an internal control. Each value was expressed as mean ± standard deviation (SD) of three experiments. Statistical significance was assigned as ** *p* < 0.01 and *** *p* < 0.001 compared to untreated control cells. ^#^ *p* < 0.05, ^##^ *p* < 0.01 and ^###^ *p* < 0.001 compared to FKB alone treated cells.

We further evaluated the role of ROS in apoptotic and autophagy cell death. For this, cells were pretreated with 5 mM NAC for 30 min and then followed by exposure to different concentrations of FKB (0–10 µg/mL) for A375 cells and 0–15 µg/mL for A2058 cells for 24 h. After 24 h, the cells were subjected to an MTT assay. The data showed that preventing the production of ROS by NAC indicated significant inhibition of FKB-induced cell death in A375 and A2058 cells (Figure 10A,B). This strongly suggested that FKB-induced excessive ROS eventually led to cell death in melanoma cells. Furthermore, NAC pretreatment had substantially downregulated the FKB-induced LC3-II accumulation, caspase-3 activation, and BRAF expression in A375 cells (Figure 10C). These observations demonstrate that FKB induced ROS-mediated apoptotic and autophagy cell death mechanisms in melanoma cells. 

### 2.9. Inhibition of FKB-Induced Apoptosis Suppressed Autophagy

**Figure 11:**
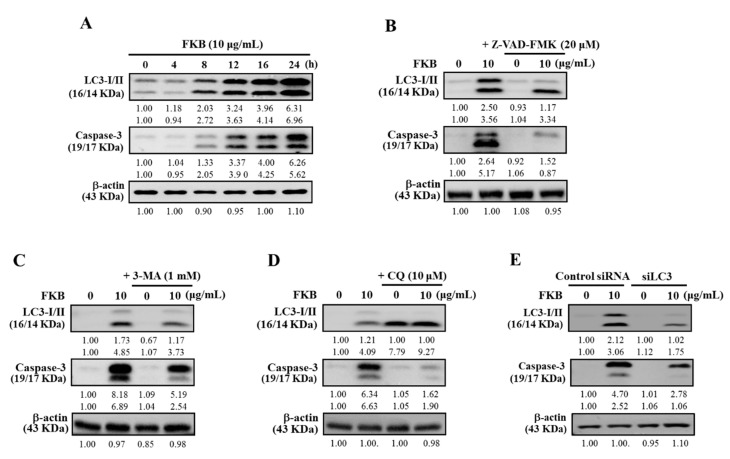
Inhibition of either apoptosis or autophagy compromised occurrence of FKB-induced cell death in A375 cells. (**A**) Western blot showing the effect of time on the expression patterns of LC3-I/II and caspase-3 with response to FKB (10 μg/mL) for 0–24 h. (**B**–**D**) Western blot showing the expression of LC3-I/II and caspase-3 levels in different conditions. (**B**) Cells were pretreated with caspase inhibitor (Z-VAD-FMK, 20 μM) and (**C**,**D**) early or late autophagy inhibitors (3-MA, 1 mM and CQ, 10 μM) for 1 h followed by FKB (10 μg/mL) for 24 h. (**E**) Control small interfering RNA (siRNA) and LC3 knockdown cells (siLC3) were treated with FKB (10 μg/mL) for 24 h, followed by the measurement of the expressions of LC3-I/II and caspase-3 proteins through the Western blot. β-actin was used as an internal control. Results are expressed as the mean ± standard deviation (SD) of three experiments.

The activation of caspases is central to any occurrence of apoptosis. In different conditions, autophagy can sometimes come earlier and activate apoptosis via caspase dependent manner and vice-versa [45]. Therefore, inhibition of either one of these mechanisms would suppress the other. First, the effect of FKB on the expression patterns of key marker proteins associated with autophagy (LC3-I/II) and apoptosis (caspase-3) were tested. Figure 11A shows 10 μg/mL FKB time-dependently and simultaneously increased the expressions of both LC3-I/II and caspase-3 proteins in A375 cells, suggesting the occurrences of autophagy and apoptosis in A375 cells. To reveal the interdependent effects of FKB-induced autophagy and apoptosis, the pharmacological inhibitor against apoptosis (Z-VAD-FMK) was used. A375 cells pretreated with an apoptotic inhibitor, Z-VAD-FMK (20 μM), inhibited the FKB-induced caspase-3 activation. This inhibition was also linked with the suppression of LC3-I/II expression in A375 cells, indicating the attenuation of FKB-induced autophagy (Figure 11B).

### 2.10. Attenuation of Apoptosis Due to the Inhibition of FKB-Induced Autophagy

To further demonstrate the effect of pharmacological inhibition of FKB-induced autophagy on the apoptosis mechanism, the inhibitors of both early and late autophagy (3-MA/CQ) as well as the silencing of LC3 methods were used. Western blot data indicated that A375 cells pretreated with the inhibitors of early (1 mM, 3-MA) or late (10 μM, CQ) autophagy inhibited the FKB-induced LC3-II accumulation. This effect was also associated with the attenuation of caspase-3 expression as well (Figure 11C,D). Similar to the autophagy inhibition, LC3 silencing was unable to convert the LC3-I to LC3-II, and caspase-3 activation was reduced even in the presence of FKB in LC3 knockdown cells (Figure 11E). All these data suggest that the inhibition of autophagy resulted in the attenuation of FKB-induced apoptosis in A375 cells.

### 2.11. FKB Treatment Inhibited the Tumor Growth in A375 Xenografted Athymic Nude Mice In Vivo

**Figure 12:**
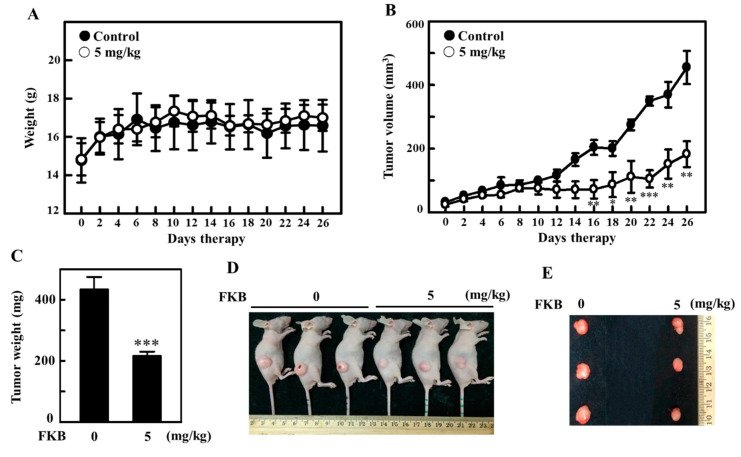
FKB-mediated in vivo inhibition of tumor growth in A375 xenograft athymic nude mice. Athymic nude mice were subcutaneously xenografted with A375 cells as described in the methods section. Mice were subjected to the vehicle (control) or FKB (5 mg/kg, intraperitoneal) for 26 days of therapy. (**A**) The body weight (in grams) and (**B**) tumor volumes (in mm^3^) were measured every 2 days. (**C**–**E**) On the twenty sixth day, animals were photographed, sacrificed and their tumor tissues were removed and weighed. Results are expressed as the mean ± standard deviation (SD) of three experiments. Statistical significance was assigned as * *p* < 0.05, ** *p* < 0.01, and *** *p* < 0.001 as compared to vehicle-treated control group.

The anti-tumor properties of FKB in vivo was tested in athymic nude mice. A375 cells were subcutaneously xenografted on nude mice as described in the methods section of this paper. The change in body weight, tumor volume and tumor weights was periodically measured and used to determine the effect of FKB’s anti-tumor properties in vivo. With increasing time, all mice were found to be healthy and no loss in body weight was noted during FKB (5 mg/kg) treatment (Figure 12A). However, with the time course, the effect of intraperitoneal FKB treatment on A375-xenograft tumor growth resulted in the inhibition of tumor volume (Figure 12B). After 26 days of therapy, the xenograft tumors excised from the mice were further evaluated and it was revealed that intraperitoneal administration of FKB treatment resulted in a dramatic retardation of tumor growth, signifying in vivo anti-tumor properties for FKB (Figure 12C–E).

## 3. Discussion

Higher incidence of melanoma is observed in Caucasian populations [46]. Rising evidence indicates that natural substances that can inhibit the cell proliferation or induce apoptosis are promising cancer treatment strategies, including melanoma cancer [47]. In this study, FKB substantially inhibited cell survival and the growth of different cell lines. In comparison to the other cell lines, A375 melanoma cells exhibited the lowest IC_50_ value of 7.16 µg/mL and 10 µg/mL FKB decreased cell proliferation and growth significantly. However, as demonstrated in our previous study [48], FKB has been found to show less cytotoxicity in normal cells too (Figure 1C–D). Nevertheless, further analysis should be done to determine FKB’s effect on normal cell lines.

FKB caused sharp changes in the morphology of melanoma cells, indicating its anti-tumor properties. Several constitutive active kinase mutants within the microtubule-associated protein (MAP) kinase pathway have been involved in oncogenesis. One of the most predominant oncogenic mutants is BRAF (V600E). The A375 endogenously expresses BRAF and leads to the constitutive activation of the MAP kinase pathway and inhibited ERK phosphorylation in the absence of ligands [49,50]. Notably, the BRAF protein related to oncogenesis was also dose-dependently downregulated along with its downstream phosphorylated ERK1/2 proteins in BRAF-expressing A375 cells, indicating that FKB plays a critical role against oncogenesis in melanoma cells.

Growing evidence has suggested that activation of apoptosis or autophagy by phytochemical agents show some promising interventions for the prevention of cancers [51,52]. FKB induced morphological aberrations were characterized by cell shrinkage, rounded shape and blebbing of plasma membrane, which emphasized that FKB can induce apoptotic or autophagic cell death in these cells. The levels of expression of PARP, caspase-3, and dysregulated ratio of Bax/Bcl-2 showed that FKB significantly induced the apoptotic cell death mechanism in melanoma cells with the caspases playing a crucial role. MTT data showed that when melanoma cells were pretreated with Z-VAD-FMK, a caspase inhibitor, the FKB-mediated apoptotic cell death was significantly downregulated. Additionally, inactivation of caspases also attenuated melanoma cell membrane morphology. Further, annexin V-FITC and PI staining in A375 cells revealed that FKB dose-dependently elevated the percentage of early and late apoptotic cells. These observations were consistent with one of our previous studies, which focused on the anti-oral cancer properties of FKB in HSC-3 cells [53].

The microtubule-associated proteins (MAPs) family includes LC3, which is considered to be the best homologous of mammalian autophagy-related protein 8 (ATG8) and has an important role in autophagy [54]. One of the important events for the formation of autophagosomes is the conversion of LC3-I into lipidated LC3-II and localization of LC3-II in autophagosomes [55,56]. Additionally, it is an autophagosomal marker that reflects lysosomal turnover and autophagic activity. FKB increased the transformation of LC3-I into LC3-II in a dose-dependent manner. p62 (SQSTM1) is a traditional multifunctional receptor protein, which is located throughout the cell in many signaling pathways and involved in the proteasomal degradation of ubiquitinated proteins [57]. The increased p62/SQSTM1 (sequestosome 1) is associated with impaired autophagy [40]. Our experimental data demonstrated that FKB increased the expression of p62 in a dose-dependent manner. Supporting the protein data, fluorescence data also showed FKB treatment significantly increased LC3 levels (~7-fold) in melanoma cells when compared with untreated cells. Additionally, FKB significantly upregulated the formation of AVOs represented by red fluorescence, which led to the activation of autophagic influx in melanoma cells. The pharmacological inhibitors of autophagy were used in order to evaluate the role of FKB-induced autophagy in melanoma cells. Fluorescence data indicated that pretreatment with 3-MA suppressed the accumulation of LC3-II in the early stages of autophagy, whereas CQ resulted in the significant formation of AVOs, confirming that FKB induced autophagic influx in melanoma cells. Regardless of the differential effect on AVO formation, 3-MA and CQ significantly reversed FKB-induced cell death in melanoma cells as shown in MTT assay, confirming that FKB-mediated the autophagy signaling cascades as a cell death mechanism. Homeostasis and the relation between the proteins Beclin-1 and Bcl-2 is a dynamic phenomenon that decides its cellular destiny. Bcl-2 binding with Beclin-1 has been reported to decrease Beclin-1 pro-autophagic ability. Conversely, Beclin-1 does not neutralize Bcl-2′s apoptotic role [10,42]. From this, the protein data showed that FKB treatment reduced the expressions of Beclin-1 and Bcl-2 proteins dose-dependently and led to autophagy in melanoma cells.

Autophagy is regulated by the AKT/mTOR signaling pathway [43]. Pharmacological induction of autophagy through mTOR inhibition has a therapeutic effect as well as potential for prevention of cancer [58]. Based on this idea, the effect of FKB on the expression of AKT (Ser437) and mTOR (Ser2448) was demonstrated and we found that 10 µg/mL FKB treatment significantly downregulated mTOR expression, inferring increased autophagy in FKB activated melanoma cells.

The cytotoxic effects of FKB on melanoma cell lines are associated with the overproduction of intracellular ROS and dysfunction of mitochondria. When produced in low concentration, ROS are not harmful and participate in redox signaling, which leads to the usual cellular functions, adaptation, and disease prevention [59]. We demonstrated the role of ROS in FKB-mediated cell death mechanisms. The DCF fluorescence data indicated that FKB time-dependently increased the production of intracellular ROS with maximum production at 30 min after FKB treatment. However, pretreatment with NAC (5 mM), the ROS inhibitor, has significantly reduced this effect. Nakazato et al., suggested that catechins had induced ROS and caused apoptosis of various human malignant B cells [60]. Erlank et al., reported that a little concentration of polyphenols can produce H_2_O_2_, which activates Nrf2 signaling in arterioles and capillaries [61]. We successfully demonstrated the role of ROS in apoptosis induction and autophagy-mediated cell death in melanoma cells. Cell viability data showed that NAC pretreatment inhibited the FKB-induced ROS production, which further reduced the FKB-induced cell death, proposing that FKB plays an important role in apoptosis and autophagy by mediating ROS in melanoma cells. Since FKB-induced ROS activated autophagy and apoptosis, its inhibition by NAC contributed to the suppression of autophagy and apoptosis. The data also indicated that LC3-II, caspase-3, and BRAF proteins were suppressed by NAC pretreatment.

Traditionally, apoptosis and autophagy were seen as slightly different forms of a death mechanism. In addition, autophagy as a response to anticancer drugs tends to be a pro-survival mechanism characterized by pro-apoptotic signals that lead to cell death [41,62]. Despite various chemotherapeutic agents that actively trigger autophagy in different cancer cell types, the role played by autophagy either in cell survival or cell death remains unclear [63,64]. Maiuri et al., indicated that inhibiting important proteins associated with apoptosis or autophagy mechanisms would be able to enhance autophagy or cause a stress response in cells from the default apoptotic pathway into increased autophagy [11]. Based on this, the cross-talk between FKB-induced autophagy and apoptosis in melanoma cells were demonstrated. Caspase-3 and LC3-II marker proteins for apoptosis and autophagy, respectively, were shown to be time-dependently expressed with a maximum expression at 24 h indicating that FKB can induce both apoptosis and autophagy. It is notable to mention that pharmacological inhibition of apoptosis (using Z-VAD-FMK) not only inhibited the expression of FKB-induced caspase-3 activation, but also accumulated LC3-II in the same cells, signifying the attenuation of FKB-induced autophagy. Similar to this, pharmacological inhibitors of both early (3-MA) and late autophagy (CQ) suppressed the expression of LC3-II protein along with the attenuation of caspase-3 protein, an apoptotic protein. Autophagic inhibition has been reported to trigger apoptosis as revealed by rising levels of caspase-3 in breast cancer cells [41]. This suggests that caspases are involved in the intervention of apoptosis and autophagy either for the death or for survival of cancer cells. More supporting evidence was provided by the inhibition of autophagy (3-MA and CQ) or silencing of LC3 expression in melanoma cells. Protein expression showed that the silencing of LC3 was not able to change LC3-I to LC3-II, which also suppressed the caspase-3 activation even in the presence of FKB. These data provided strong support that there is a cross-talk between FKB-induced apoptosis and autophagy in melanoma cells. The exact molecular phenomenon that can trigger autophagy and apoptosis needs to be highlighted in the literature. There is some evidence from the literature that autophagic induction and/or apoptotic responses to cellular stresses are characterized as serial, concurrent, or mutually exclusive processes [65]. Our in vitro study showed consistency with an in vivo study, which revealed that the size of tumor in A375 xenografted nude mice was significantly reduced by FKB treatment. FKB successfully enhances apoptosis and autophagy in A375 human melanoma cells. 

## 4. Materials and Methods 

### 4.1. Reagents 

Glutamine, Dulbecco’s modified Eagle’s medium (DMEM), penicillin/streptomycin, and fetal bovine serum (FBS) were purchased from GIBCO BRL (Grand Island, NY, USA). Acridine orange, 3-(4,5-dimethylthiazol-2-yl)-2,5-diphenyltetrazolium bromide (MTT), 3-methyladenine (3-MA), propidium iodide (PI), *N*-acetylcysteine (NAC), chloroquine (CQ), and 2′-7′dichlorofluorescin diacetate (DCFH_2_-DA) were acquired from Sigma-Aldrich (St. Louis, MO). Z-Val-Ala-Asp fluoromethyl ketone (Z-VAD-FMK) was purchased from Calbiochem (San Diego, CA, USA). Antibody against caspase-9 was purchased from Thermo Fisher Scientific, Inc. (Waltham, MA, USA). Antibodies against β-actin, p-ERK1/2, BRAF, Bax, p-AKT, and Bcl-2 were obtained from Santa Cruz Biotechnology, Inc. (Heidelberg, Germany). Antibodies against Beclin-1, SQSTM1/p62, p-mTOR, PARP, caspase-3, and LC3-I/II were acquired from Cell Signaling Technology, Inc. (Danvers, MA, USA). All secondary antibodies were obtained from Santa Cruz Biotechnology (Santa Cruz, CA) (for more information, please check Supplementary Table S1). All other chemicals were bought from Merck and Co., Inc. (Darmstadt, Germany) or Sigma-Aldrich (St. Louis, MO, USA). 

### 4.2. FKB Treatment

FKB was bought from LKT Laboratories., Inc. (St. Paul, MN, USA) (product ID. F4503). As confirmed by HPLC analysis, over 99% pure FKB was used for the study. FKB (10 mg/mL or 35.2 mM) of stock solution was prepared in 100% DMSO. The stock solution was diluted in medium when necessary in such a way that the final concentration of DMSO remained 0.1%. 

### 4.3. Cell Culture

Human epithelial melanoma cell line (A375), human skin lymph node derived melanoma cell line (A2058 [66]), human primary epidermal melanocyte cell line (HEMn), and human skin keratinocyte cell line (HaCaT) cells were purchased from American Type Culture Collection (ATCC, Rockville, MD, USA). All cells were cultured in DMEM supplemented with 2 mM glutamine, 10% FBS and 1% penicillin/streptomycin and maintained in 5% CO_2_ at 37 °C. Cell numbers were determined using hemocytometer, and cell morphology was determined using phase-contrast microscopy. 

### 4.4. Cell Viability Assay (MTT) and Determination of IC_50_ Value of FKB

MTT was used to determine the cell viability [47]. Briefly, 5 × 10^4^ cells/well were grown in a 24-well plate and were exposed to various concentrations of FKB (0–20 µg/mL) for 24 h. After treatments, the supernatant was removed and replaced with 400 μL of 0.5 mg/mL MTT and then incubated at 37 °C for 2 h. MTT formazan crystals were dissolved in equal volumes in 90% isopropanol and 0.5% SDS mixture and at 570 nm (A_570_), absorbance was recorded with an ELISA microplate reader (µ-Quant, Winooski, VT, USA). The following formula was used to calculate the percentage (%) of cell viability: (A_570_ of treated cells/A_570_ of untreated cells) × 100. The cytotoxic effects of FKB were expressed as IC_50_. 

### 4.5. Protein Isolation and Western Blot

For protein isolation, a 10-cm dish was taken with 1 × 10^6^ cells that were incubated in the presence or absence of FKB (0–10 μg/mL) for 0–24 h. PBS washed cells were then suspended in 100 μL of lysis buffer containing 10 mM tris-HCl, pH 8.0, 320 mM sucrose, 1% Triton X-100, 1 mM PMSF, 2 mM DTT, and 5 mM EDTA. Cells were placed on ice for 20 min and then centrifuged at 15,000×*g* for 30 min at 4 °C. Total protein concentration was determined with a Bio-Rad protein assay reagent (Bio-Rad, Hercules, CA, USA) with BSA as standard for protein estimation. The protein extracts were denatured by sample buffer containing 62 mM tris-HCl, 2% SDS, 10% glycerol, and 5% β-mercaptoethanol. Then, the mixture was boiled at 97 °C for 5 min. Equal quantities (50 μg) of denatured protein samples were separated on a gradient SDS-PAGE gel (8–18%) and transferred onto polyvinylidene difluoride (PVDF) membranes. Non-specific protein bindings were prevented from blocking the membranes with 5% blotto (5% non-fat dried milk, PBS containing 1% Tween-20) for 1 h at room temperature. Later, the blocked membranes were exposed to various primary antibodies overnight. The next day, primary antibodies were retained, and membranes were incubated with either horseradish peroxidase (HRP)-conjugated anti-rabbit or anti-mouse antibodies for 2 h at room temperature. Immuno-reactive bands were visualized through a chemiluminescent substrate (Millipore, Billerica, MA, USA) and changes in protein levels were digitalized using the ImageQuant™ LAS 4000 mini (Fujifilm). Densitometric analysis was performed with AlphaEase quantitative software (Genetic Technology Inc. Miami, FL, USA). Data were shown as fold difference over untreated control. The internal controls were β-actin and histone proteins for total and nuclear proteins, respectively [67].

### 4.6. Detection of Apoptotic Cells by Annexin V and PI Staining

Staining with annexin V-fluorescein isothiocyanate (FITC) and propidium iodide (PI) was done to probe and detect apoptotic rates in melanoma cells. In brief, different concentrations of FKB (0–10 μg/mL) were used to treat cells for 24 h. Later, the cells were trypsinized, washed twice with PBS, and centrifuged at 800 rpm for 5 min. Approximately, 1 × 10^6^ cells were placed in 0.5 mL of binding buffer and double-stained with annexin V-FITC and PI for 15 min at room temperature. The resultant red (PI) and green (FITC) fluorescence emissions for each sample were quantitatively measured with a FACSCaliber flow cytometer (Becton Dickinson, San Jose, CA, USA) and analyzed using Cell Quest software. Based on the staining properties of the cells, the results obtained were categorized as; (Q1) necrotic cells: annexin V-FITC negative, PI positive; (Q2) apoptotic cells: annexin V-FITC positive, PI positive; (Q3) normal live cells: annexin V-FITC negative, PI negative; and (Q4) early apoptotic cells: annexin V-FITC positive, PI negative cells [47].

### 4.7. Detection and Quantification of AVOs with Acridine Orange Staining

Acridine orange (AO) is a weak lysosomotropic metachromatic fluorochrome [68]. The cells were incubated for 24 h with the indicated concentration of FKB in the absence or presence of 3-MA (a PI3K inhibitor, 1mM), Z-VAD-FMK (caspase inhibitor, 20 μM), NAC (ROS inhibitor, 1 mM), and CQ (autophagic inhibitor, 10 μM) for 1 h. After treatment, cells were washed with PBS and stained with AO (1 μg/mL in PBS + 5% FBS) for 15 min. AVOs formed in cells were observed with a fluorescence microscope using a red filter at 100× magnification. AO’s fluorescence emission is concentration dependent. Red (lysosomes), green (cytosol), and yellow emissions showed low, medium, and high concentrations, respectively [31]. 

### 4.8. LC3 Immunofluorescence

Approximately, 10^4^ A375 cells were grown in each well of an 8-well Lab-Tek chamber (Thermo Fisher Science, Waltham, MA, USA) in DMEM with 10% FBS. These cells were pretreated with FKB (0–10 μg/mL, 24 h) and fixed for 15 min in 2% paraformaldehyde and permeabilized for 10 min with 0.1% Triton X-100. Later, cells were washed and blocked in PBS with 10% FBS followed by incubation in 1.5% FBS with a primary antibody LC3B for 2 h. In 6% BSA, a secondary FITC (488 nm) antibody was incubated for another 1 h. The cell nuclei were stained for 5 min with 4′,6-diamidino-2-phenylindole (DAPI) (1 μg/mL). The stained cells were washed with PBS and visualized (100× magnification) using a fluorescence microscope [35].

### 4.9. siRNA Targeting LC3 Transfection

The key biomarker to show autophagy in mammalian systems is LC3 [69]. For transfection, 2.5 × 10^5^ cells were cultured in each well of a 6-well plate. When cell confluency reached 40–60%, then the culture medium was substituted with 0.5mL of Opti-MEM (GIBCOBRL/Invitrogen). Lipofectamine RNAiMAX (Invitrogen) was used to transfect siRNA. Then, 100 pM siRNA was incubated with the Opti-MEM and 0.5 mL lipofectamine RNAiMAX reagent for approximately 25 min at room temperature to form complex. Then, the mixture was slowly added to the cells in the 6-well plate to make a transfection volume of 1 mL and was incubated for 6 h. Later, 2 mL of growth medium was substituted for the transfection medium and cultured at room temperature [70]. 

### 4.10. Intracellular ROS Measurement

A cell-permeable fluorogenic probe, 2′,7′-dihydrofluorescein-diacetate (DCFH2-DA), was used to measure the effect of FKB on intracellular ROS production in A375 cells. For this, A375 cells confluent at 80% were incubated with 10 µg/mL of FKB for 0, 15, 30, 60, and 90 min. Following the incubation, fresh medium containing 10 μM DCFH2-DA was added to the cells and incubated for 30 min at 37 °C. The levels of intracellular ROS depend on the accumulation of intracellular dichlorofluorescein (DCF), which is formed by the oxidation of DCFH2-DA. The intensity of fluorescence was measured using a fluorescence microscope [35]. 

### 4.11. Animal Care and Maintenance

In vivo experiments were performed as previously described [70]. Athymic nude BALB/*c-nu* mice (8 weeks old) were procured from the National Laboratory Animal Center (NLAC; Taipei, Taiwan). The mice used in this research were kept in a pathogen-free and specially designed animal care facility that offered 12 h of light/dark. The mice used in this research freely accessed water and rodent chow (Oriental Yeast Co Ltd., Tokyo, Japan). This research ensured that all animal protocols were reviewed and approved (approval no: 104-55-N) by Institutional Animal Care and Use Committee (IACUC) of China Medical University. All animal study protocols and methods were conducted following the “Guidelines for the Care and Use of Laboratory Animals”. The aforementioned guidelines were issued by the Chinese Society of Animal Science, Taiwan. 

### 4.12. Tumor Cell Inoculation

Ten female athymic nude mice were chosen for the in vivo studies. Approximately, 2 × 10^6^ A375 cells were mixed in 200 µL of matrix gel and injected into the subcutaneous layer of the hind flanks of each mouse. Seven days after cell inoculation, they were randomly and equally divided into two groups: control and treatment sets, with five mice in each group. Control and treatment groups received 0.1% DMSO or freshly prepared FKB in 0.1% DMSO (5 mg/kg, every two days) through intraperitoneal injections, respectively. Tumor volumes, drug toxicity, and body weight for all subjects were monitored every 2 days. Length, width, and depth of the tumor were measured every week and tumor volumes were calculated from formula, length × width^2^ × ½. On day 26, the mice used in this study were sacrificed and tumor tissues were gathered and then weighed. Additionally, all the organs were further examined by a veterinary pathologist [31,70]. 

### 4.13. Statistical Analysis

All data are shown as the mean ± standard deviation (SD) of triplicate experiments. Analysis of variance (ANOVA) was completed with Dunnett’s test for pairwise comparison of the treated group with the control group. Statistical significance between treatment groups were assigned as * *p* < 0.05, ** *p* < 0.01, and *** *p* < 0.001 as compared to the untreated control cells; whereas, ^#^ *p* < 0.05, ^##^ *p* < 0.01, and ^###^ *p* < 0.001 as compared to the treated group.

## 5. Conclusions

In conclusion, our study indicates that FKB induced melanoma cell death via both apoptotic and autophagic mechanisms in A375 and/or A2058 cells. Additionally, FKB-induced ROS seemed to be a key initiator for the transmission of apoptosis and autophagy signals. Apoptosis or autophagy signaling reduced cell death induced by FKB due to the inhibition of ROS production. FKB-induced apoptosis was displayed by increased staining of cells by annexin-V FITC/PI, Bax/Bcl-2 proportion, initiation of caspase-3, and PARP cleavage. Occurrences of autophagy were characterized by the elevated accumulation of LC3-II, increased level of SQSTM1/p62 and AVOs formation. Dysregulated Beclin-1/Bcl-2 levels and decreased AKT/mTOR signaling pathways mediated the execution of autophagy. Pharmacological inhibition of either apoptosis or autophagy suppressed other mechanisms, which indicated that there are some cross talks between FKB-mediated apoptosis and autophagy in melanoma cells. The in vivo data also provided a consistent observation in which time-dependent treatment of FKB suppressed the xenograft-induced tumor mass and volume in the nude mice. Future studies on the effects of FKB will aid the development of strategies and provide better insights into the prevention of human melanoma progression. 

## Supplementary Materials

The following are available online at https://www.mdpi.com/2072-6694/12/10/2936/s1, Table S1: List of all antibodies with their corresponding manufacturer’s information and item numbers. Figure S1F: Effect of FKB (0–10 μg/mL, 24 h) on the expression patterns of BRAF and p-ERK proteins. Figure S2A,B: The expression levels of FKB-induced activation of caspase-3 and PARP cleavage in A375 and A2058 cells. Figure S2C,D: The expression levels of apoptosis activator; Bax and inhibitor proteins; Bcl-2 in A375 and A2058 cells. Figure S2E: The effect of time on the expressions of Bax and Bcl-2 proteins in 10 μg/mL of FKB treated A375. Figures S4A and S4B: A375 and A2058 cells were treated with various concentrations of FKB (0–10 or 0–15 μg/mL, 24 h) to determine the conversion of LC3-I to LC3-II and expression of p62 proteins. Figure S6: FKB dysregulated Beclin-1, Bcl-2 ratio and suppressed p-AKT/p-mTOR expressions in melanoma cells. Beclin-1 and Bcl-2 expressions in A375 cells (S6A) and A2058 cells (S6B) and phosphorylation of AKT and mTOR proteins (S6C). Figure S10C: Measurement of LC3-I/II, caspase-3, and BRAF proteins both in the absence or presence of NAC pretreatment conditions. Figure S11A: Effect of time on the expression patterns of LC3-I/II and caspase-3 with response to FKB (10 μg/mL, 0–24 h). Figure S11B–D: Expression of LC3-I/II and caspase-3 levels in different conditions. Cells were pretreated with caspase inhibitor (Z-VAD-FMK, 20 μM) (S11B); early autophagy inhibitor (S11C); late autophagy inhibitor (S11D). Measurement of the expressions of LC3-I/II and caspase-3 proteins in LC3 knockdown cells (S11E).
