# Correction: Hseu, Y.-C., et al. The In Vitro and In Vivo Anticancer Properties of Chalcone Flavokawain B through Induction of ROS-Mediated Apoptotic and Autophagic Cell Death in Human Melanoma Cells. *Cancers* 2020, *12*, 2936

**DOI:** 10.3390/cancers13020303

**Published:** 2021-01-15

**Authors:** You-Cheng Hseu, Yu-Chi Chiang, Yugandhar Vudhya Gowrisankar, Kai-Yuan Lin, Sheng-Teng Huang, Sirjana Shrestha, Geng-Ruei Chang, Hsin-Ling Yang

**Affiliations:** 1 Department of Cosmeceutics, College of Pharmacy, China Medical University, Taichung 40402, Taiwan; ychseu@mail.cmu.edu.tw (Y.-C.H.); dr.vgyugandhar@mail.cmu.edu.tw (Y.V.G.); 2 Department of Health and Nutrition Biotechnology, Asia University, Taichung 41354, Taiwan; 3 Chinese Medicine Research Center, China Medical University, Taichung 40402, Taiwan; 4 Research Center of Chinese Herbal Medicine, China Medical University, Taichung 40402, Taiwan; 5 Institute of Nutrition, College of Biopharmaceutical and Food Sciences, China Medical University, Taichung 40402, Taiwan; u101008303@cmu.edu.tw (Y.-C.C.); sirju10@mail.cmu.edu.tw (S.S.); 6 Department of Medical Research, Chi Mei Medical Center, Tainan 71004, Taiwan; d970712@mail.chimei.org.tw; 7 Department of Biotechnology, Chia Nan University of Pharmacy and Science, Tainan 71004, Taiwan; 8 School of Chinese Medicine, China Medical University, Taichung 40402, Taiwan; sthuang@mail.cmu.edu.tw; 9 Department of Veterinary Medicine, National Chiayi University, Chiayi 60054, Taiwan

In the original article [1], there was a mistake in Figure 1E as published: Figure 1E (A2058) was a duplication of Figure 3D. The mistake was due to an unnoticed error that was overlooked during the manuscript submission and revision processes. This error is corrected by providing a new Figure 1E.

The original Figure 1E is:


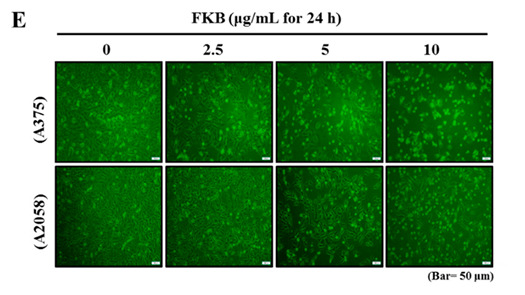


and should be replaced with the following version:


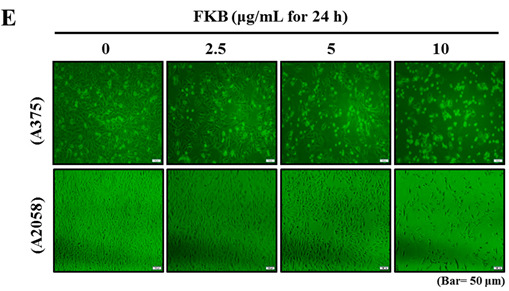


The authors apologize for any inconvenience caused and state that the scientific conclusions are unaffected. The original article has been updated.
