# Supplementary material for: The In Vitro and In Vivo Anticancer Properties of Chalcone Flavokawain B through Induction of ROS-Mediated Apoptotic and Autophagic Cell Death in Human Melanoma Cells
Source: Cancers (Basel). 2020 Oct 12;12(10):2936. doi: 10.3390/cancers12102936 (PMC7600613; doi:10.3390/cancers12102936)
Supplement: Supplementary file 1 [file cancers-12-02936-s001.zip › Suplementary Table_List of antibodies.docx]

| Name of the antibody | | Manufacturer | Item No. |
| --- | --- | --- | --- |
| B | Beclin-1 | Cell Signaling Technology | #3495 |
|  | Bcl-2 | Santa Cruz Biotechnology | sc-492 |
|  | β-actin | Santa Cruz Biotechnology | sc-47778 |
|  | Bax | Cell Signaling Technology | #2772 |
|  | BRAF | Santa Cruz Biotechnology | sc-5284 |
| C | Cleaved caspase 3 | Cell Signaling Technology | #9664 |
| L | LC3B | Cell Signaling Technology | #3868 |
| P | p-mTOR | Cell Signaling Technology | #5536 |
|  | p-PI3K | Cell Signaling Technology | #4228 |
|  | PARP | Cell Signaling Technology | #9532 |
|  | p-Akt | Cell Signaling Technology | #9271 |
|  | p-ERK | Cell Signaling Technology | #4370 |
|  | SQSTM1/P62 | Cell Signaling Technology | #8025 |
