# Supplementary material for: The In Vitro and In Vivo Anticancer Properties of Chalcone Flavokawain B through Induction of ROS-Mediated Apoptotic and Autophagic Cell Death in Human Melanoma Cells
Source: Cancers (Basel). 2020 Oct 12;12(10):2936. doi: 10.3390/cancers12102936 (PMC7600613; doi:10.3390/cancers12102936)
Supplement: Supplementary file 1 [file cancers-12-02936-s001.zip › Fig-S1.pptx]

## Slide 1
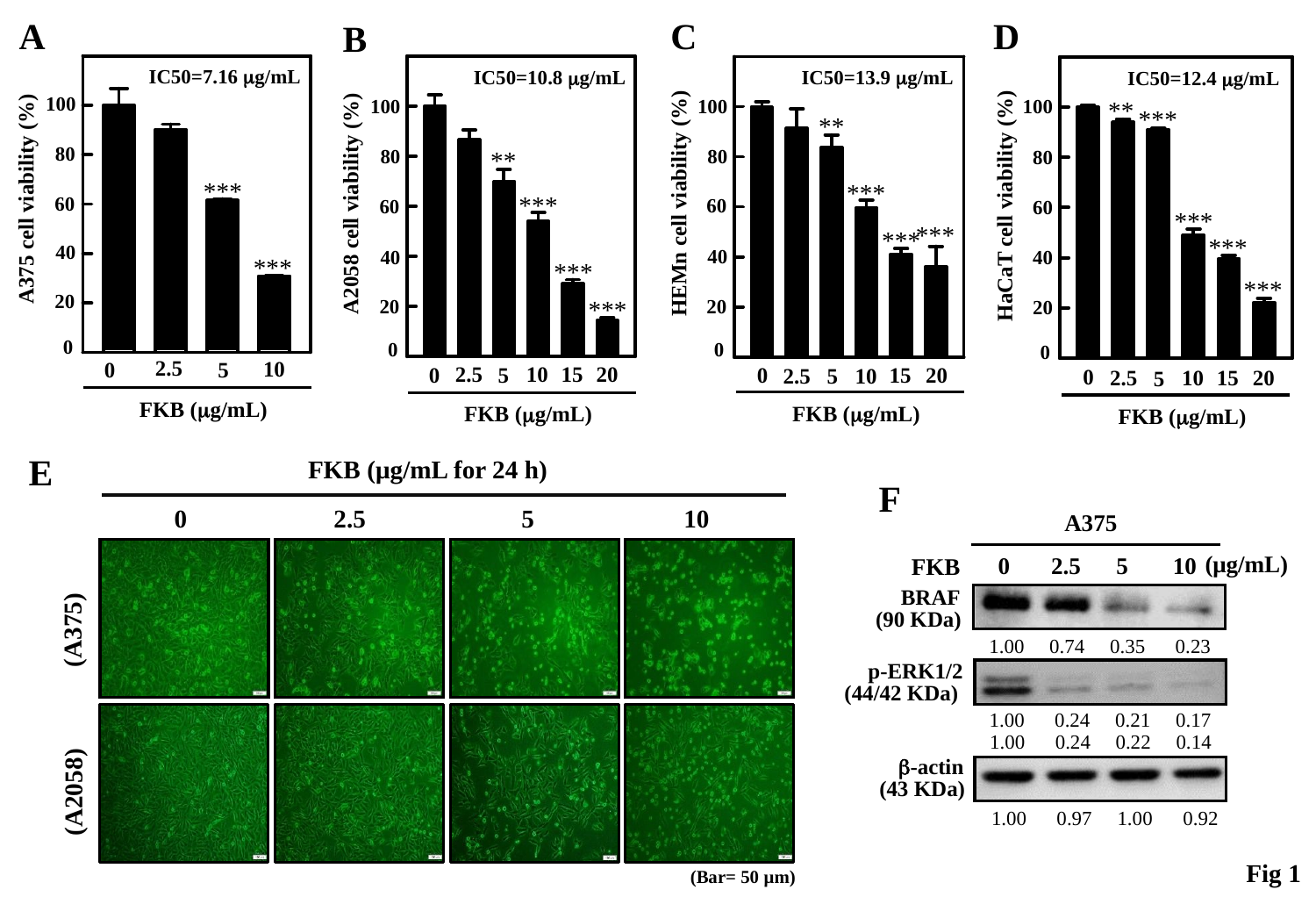

C
D
A
IC50=7.16 mg/mL
100
80
***
A375 cell viability (%)
60
40
***
20
0
2.5
10
0
5
FKB (mg/mL)
B
IC50=12.4 mg/mL
100
**
***
80
HaCaT cell viability (%)
60
***
***
40
***
20
0
0
2.5
10
15
20
5
FKB (mg/mL)
IC50=13.9 mg/mL
100
**
80
***
HEMn cell viability (%)
60
***
***
40
20
0
15
20
0
5
2.5
10
FKB (mg/mL)
IC50=10.8 mg/mL
100
80
**
***
A2058 cell viability (%)
60
40
***
***
20
0
2.5
10
15
20
0
5
FKB (mg/mL)
E
FKB (μg/mL for 24 h)
0
2.5
5
10
(A375)
(A2058)
(Bar= 50 μm)
F
A375
 (μg/mL)
 0 2.5 5 10
FKB
BRAF
(90 KDa)
1.00 0.74 0.35 0.23
p-ERK1/2
(44/42 KDa)
1.00 0.24 0.21 0.17
1.00 0.24 0.22 0.14
b-actin
(43 KDa)
1.00 0.97 1.00 0.92
Fig 1

## Slide 2
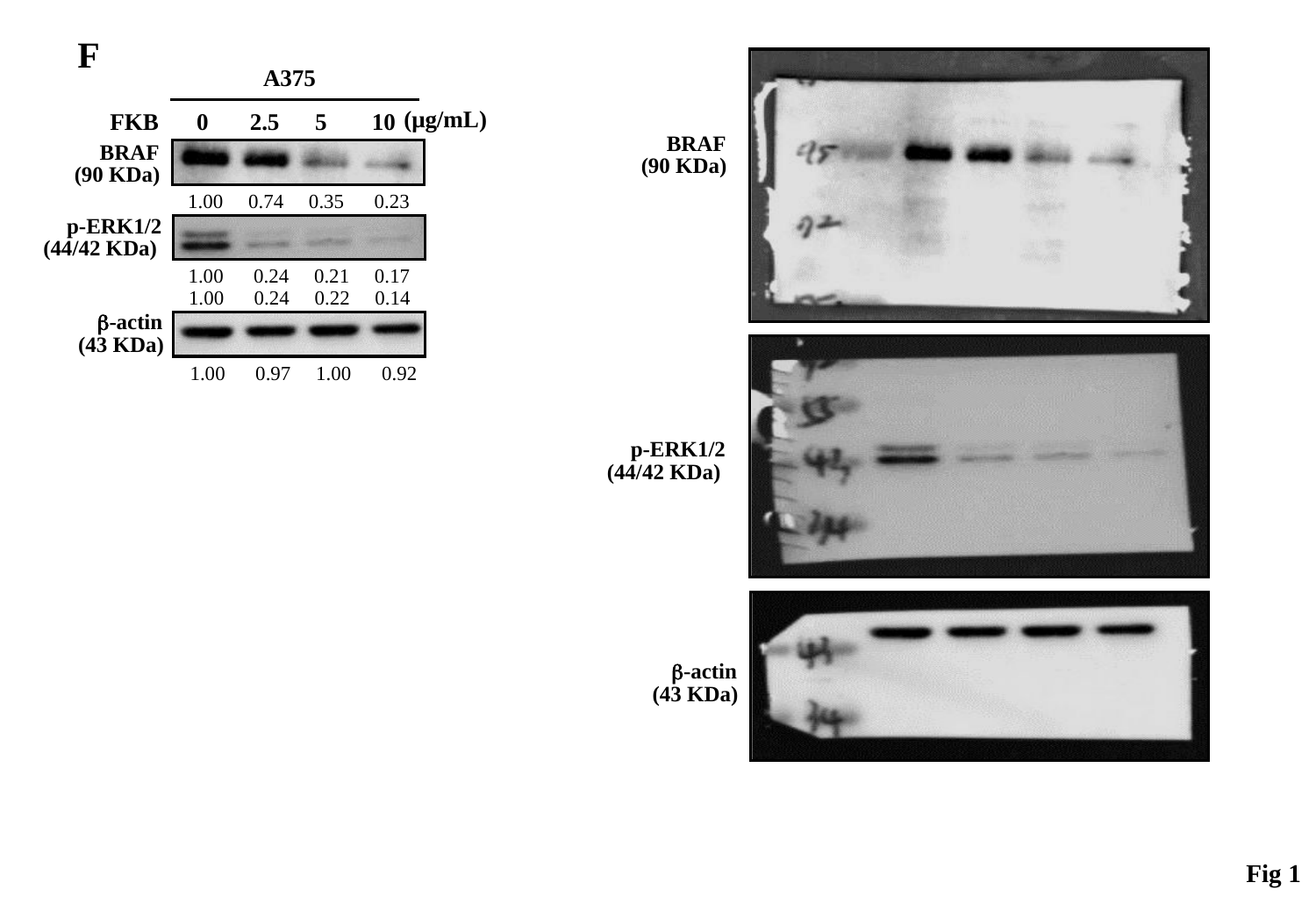

F
A375
 (μg/mL)
 0 2.5 5 10
FKB
BRAF
(90 KDa)
1.00 0.74 0.35 0.23
p-ERK1/2
(44/42 KDa)
1.00 0.24 0.21 0.17
1.00 0.24 0.22 0.14
b-actin
(43 KDa)
1.00 0.97 1.00 0.92
BRAF
(90 KDa)
p-ERK1/2
(44/42 KDa)
b-actin
(43 KDa)
Fig 1
