# Supplementary material for: The In Vitro and In Vivo Anticancer Properties of Chalcone Flavokawain B through Induction of ROS-Mediated Apoptotic and Autophagic Cell Death in Human Melanoma Cells
Source: Cancers (Basel). 2020 Oct 12;12(10):2936. doi: 10.3390/cancers12102936 (PMC7600613; doi:10.3390/cancers12102936)
Supplement: Supplementary file 1 [file cancers-12-02936-s001.zip › Fig-S2.pptx]

## Slide 1
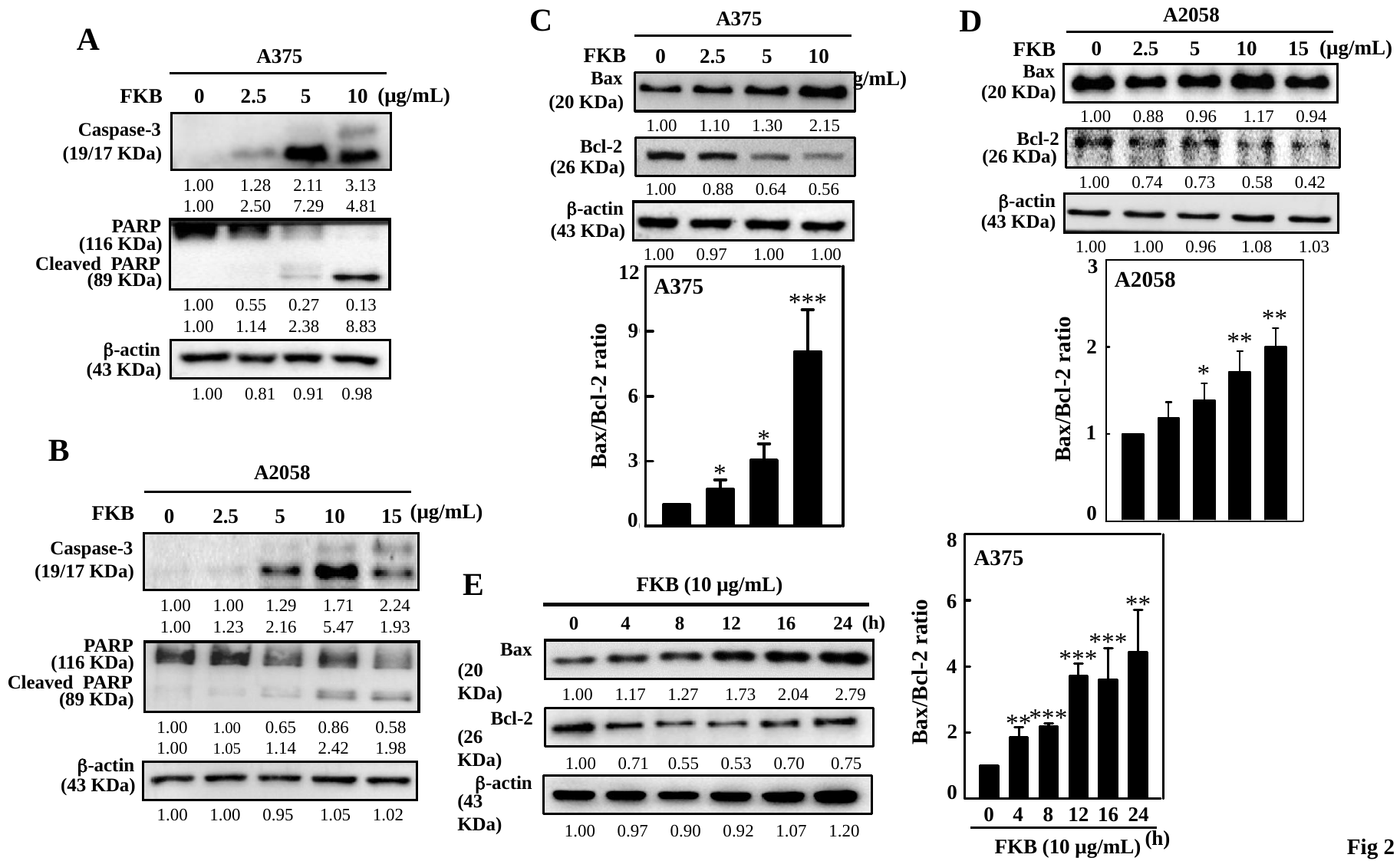

C
D
A2058
 (μg/mL)
 0 2.5 5 10 15
FKB
Bax
(20 KDa)
1.00 0.88 0.96 1.17 0.94
Bcl-2
(26 KDa)
1.00 0.74 0.73 0.58 0.42
b-actin
(43 KDa)
1.00 1.00 0.96 1.08 1.03
3
A2058
**
**
2
*
Bax/Bcl-2 ratio
1
0
A375
A
A375
(μg/mL)
FKB
 0 2.5 5 10
Caspase-3
(19/17 KDa)
1.00 1.28 2.11 3.13
1.00 2.50 7.29 4.81
PARP
(116 KDa)
Cleaved PARP
(89 KDa)
1.00 0.55 0.27 0.13
1.00 1.14 2.38 8.83
b-actin
(43 KDa)
1.00 0.81 0.91 0.98
 (μg/mL)
FKB
 0 2.5 5 10
Bax
(20 KDa)
1.00 1.10 1.30 2.15
Bcl-2
(26 KDa)
1.00 0.88 0.64 0.56
b-actin
(43 KDa)
1.00 0.97 1.00 1.00
12
A375
***
9
Bax/Bcl-2 ratio
6
*
B
A2058
(μg/mL)
FKB
 0 2.5 5 10 15
Caspase-3
(19/17 KDa)
1.00 1.00 1.29 1.71 2.24
1.00 1.23 2.16 5.47 1.93
PARP
(116 KDa)
Cleaved PARP
(89 KDa)
1.00 1.00 0.65 0.86 0.58
1.00 1.05 1.14 2.42 1.98
b-actin
(43 KDa)
1.00 1.00 0.95 1.05 1.02
3
*
0
8
A375
6
**
***
***
4
Bax/Bcl-2 ratio
***
***
**
2
0
 (h)
4
12
0
8
16
24
FKB (10 μg/mL)
E
FKB (10 μg/mL)
 (h)
0
4
8
12
16
24
Bax
(20 KDa)
1.00 1.17 1.27 1.73 2.04 2.79
Bcl-2
(26 KDa)
1.00 0.71 0.55 0.53 0.70 0.75
b-actin
(43 KDa)
1.00 0.97 0.90 0.92 1.07 1.20
Fig 2

## Slide 2
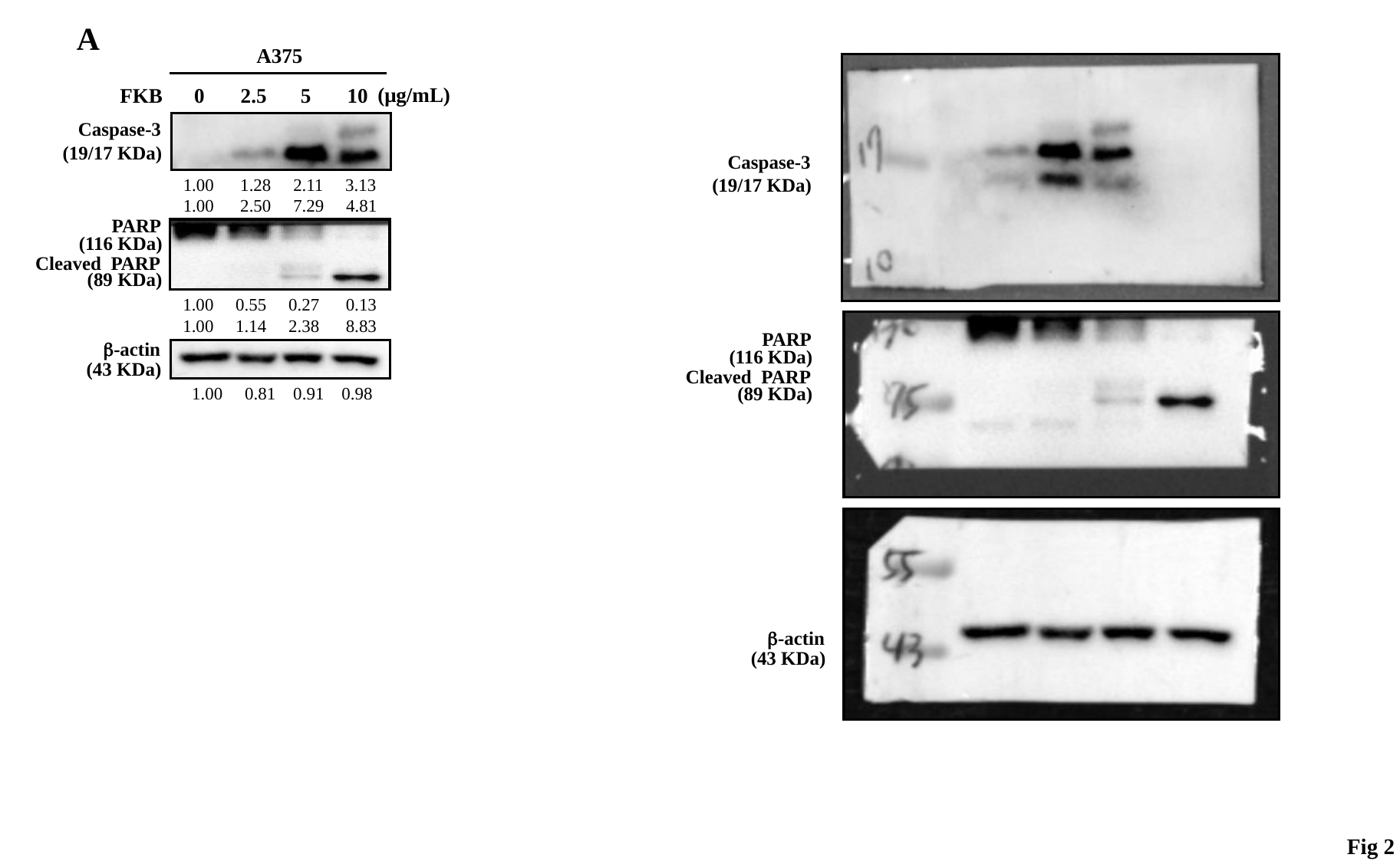

A
A375
(μg/mL)
FKB
 0 2.5 5 10
Caspase-3
(19/17 KDa)
1.00 1.28 2.11 3.13
1.00 2.50 7.29 4.81
PARP
(116 KDa)
Cleaved PARP
(89 KDa)
1.00 0.55 0.27 0.13
1.00 1.14 2.38 8.83
b-actin
(43 KDa)
1.00 0.81 0.91 0.98
Caspase-3
(19/17 KDa)
PARP
(116 KDa)
Cleaved PARP
(89 KDa)
b-actin
(43 KDa)
Fig 2

## Slide 3
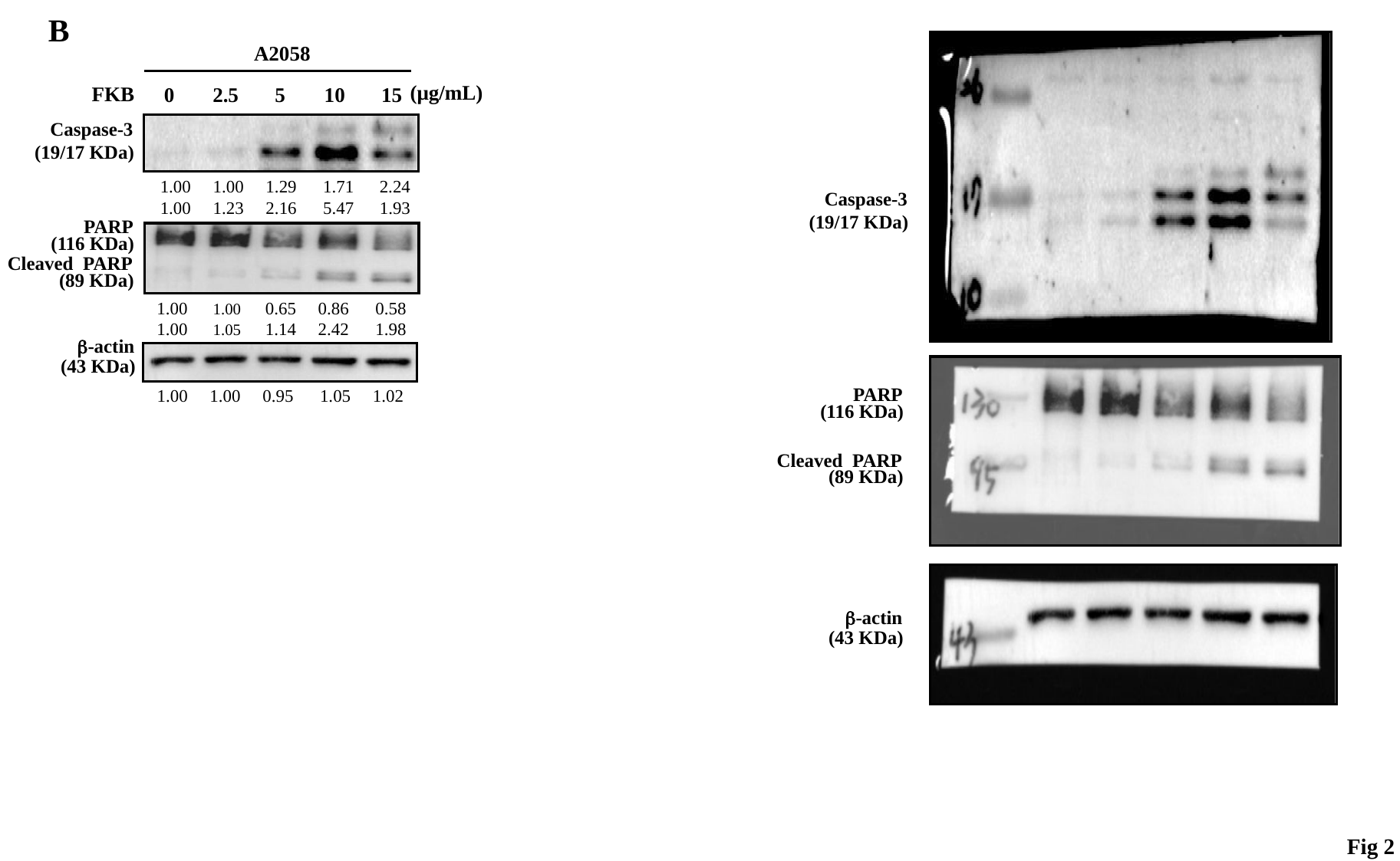

B
A2058
(μg/mL)
FKB
 0 2.5 5 10 15
Caspase-3
(19/17 KDa)
1.00 1.00 1.29 1.71 2.24
1.00 1.23 2.16 5.47 1.93
PARP
(116 KDa)
Cleaved PARP
(89 KDa)
1.00 1.00 0.65 0.86 0.58
1.00 1.05 1.14 2.42 1.98
b-actin
(43 KDa)
1.00 1.00 0.95 1.05 1.02
Caspase-3
(19/17 KDa)
PARP
(116 KDa)
Cleaved PARP
(89 KDa)
b-actin
(43 KDa)
Fig 2

## Slide 4
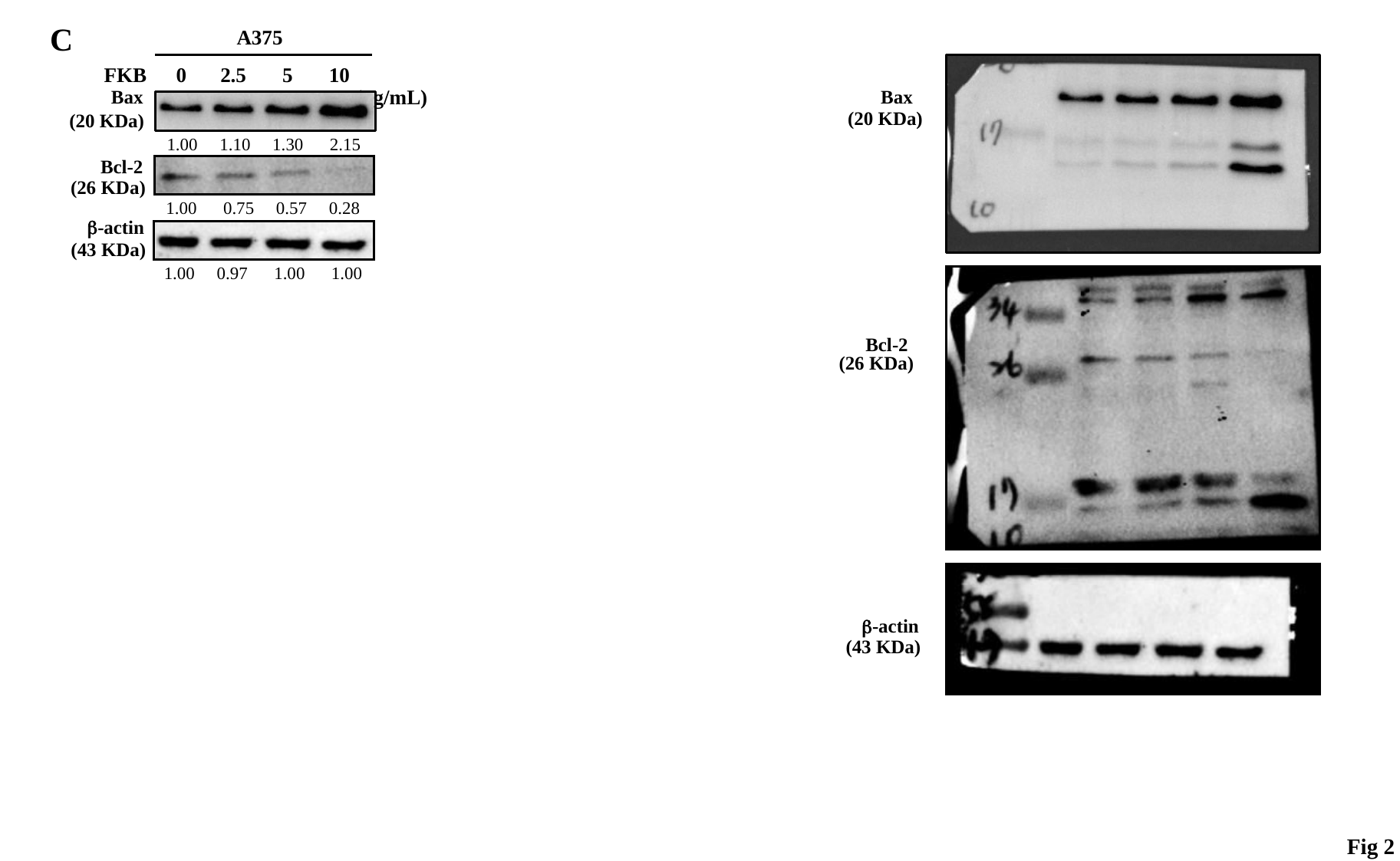

C
A375
 (μg/mL)
FKB
 0 2.5 5 10
Bax
Bax
(20 KDa)
(20 KDa)
1.00 1.10 1.30 2.15
Bcl-2
(26 KDa)
1.00 0.75 0.57 0.28
b-actin
(43 KDa)
1.00 0.97 1.00 1.00
Bcl-2
(26 KDa)
b-actin
(43 KDa)
Fig 2

## Slide 5
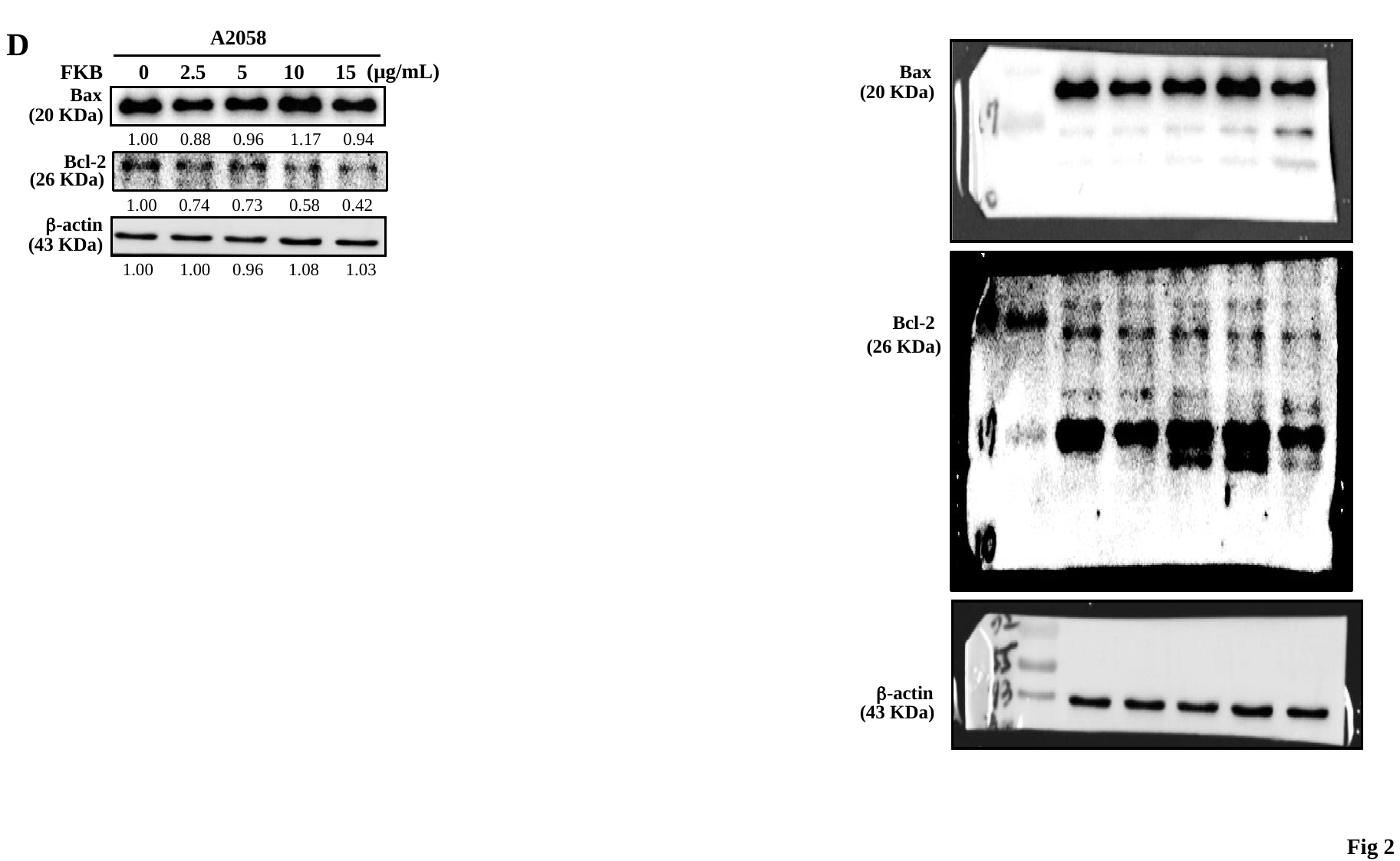

D
A2058
Bax
 (μg/mL)
 0 2.5 5 10 15
FKB
(20 KDa)
Bax
(20 KDa)
1.00 0.88 0.96 1.17 0.94
Bcl-2
(26 KDa)
1.00 0.74 0.73 0.58 0.42
b-actin
(43 KDa)
1.00 1.00 0.96 1.08 1.03
Bcl-2
(26 KDa)
b-actin
(43 KDa)
Fig 2

## Slide 6
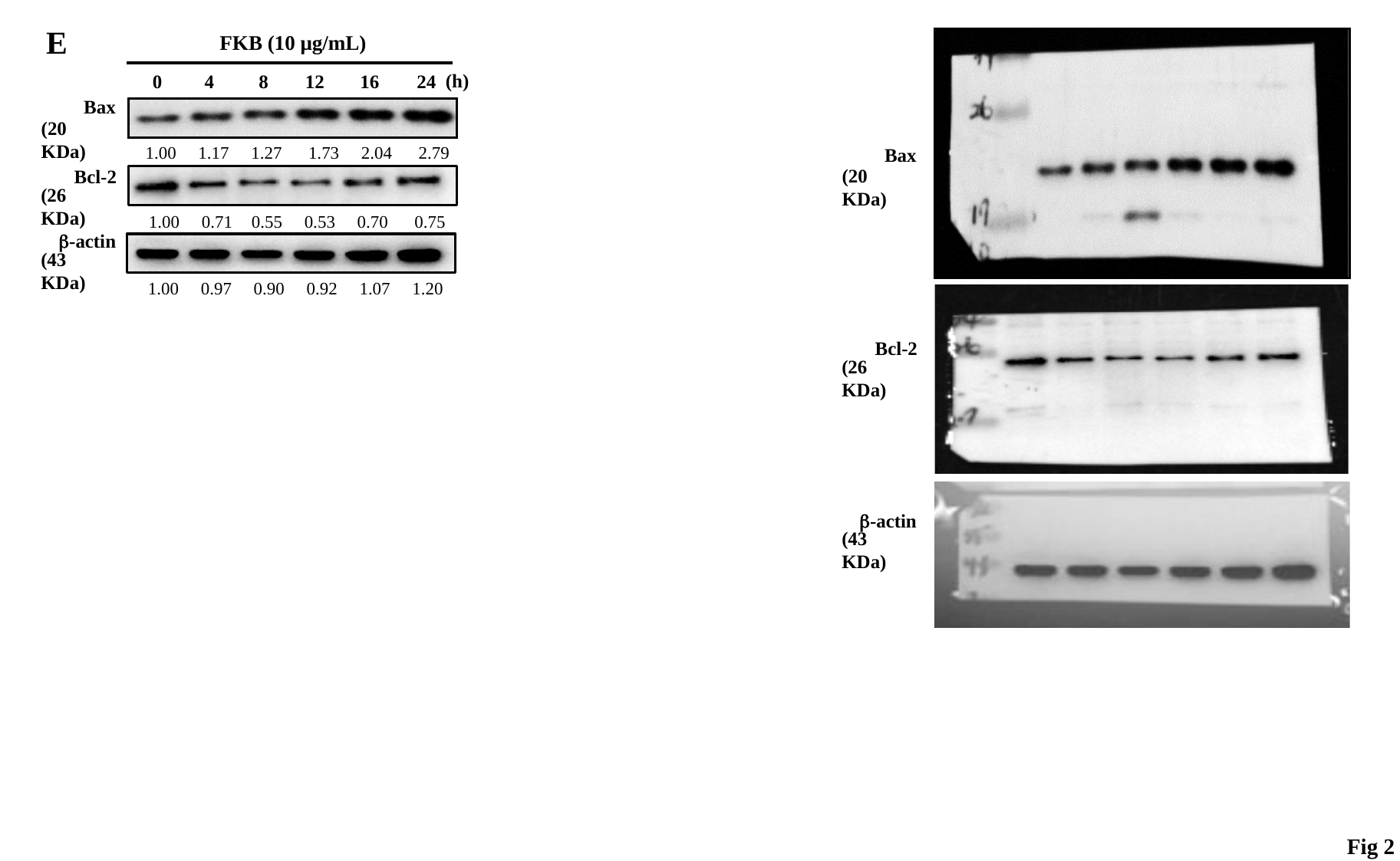

E
FKB (10 μg/mL)
 (h)
0
4
8
12
16
24
Bax
(20 KDa)
1.00 1.17 1.27 1.73 2.04 2.79
Bcl-2
(26 KDa)
1.00 0.71 0.55 0.53 0.70 0.75
b-actin
(43 KDa)
1.00 0.97 0.90 0.92 1.07 1.20
Bax
(20 KDa)
Bcl-2
(26 KDa)
b-actin
(43 KDa)
Fig 2
