# Supplementary material for: The In Vitro and In Vivo Anticancer Properties of Chalcone Flavokawain B through Induction of ROS-Mediated Apoptotic and Autophagic Cell Death in Human Melanoma Cells
Source: Cancers (Basel). 2020 Oct 12;12(10):2936. doi: 10.3390/cancers12102936 (PMC7600613; doi:10.3390/cancers12102936)
Supplement: Supplementary file 1 [file cancers-12-02936-s001.zip › Fig-S4.pptx]

## Slide 1
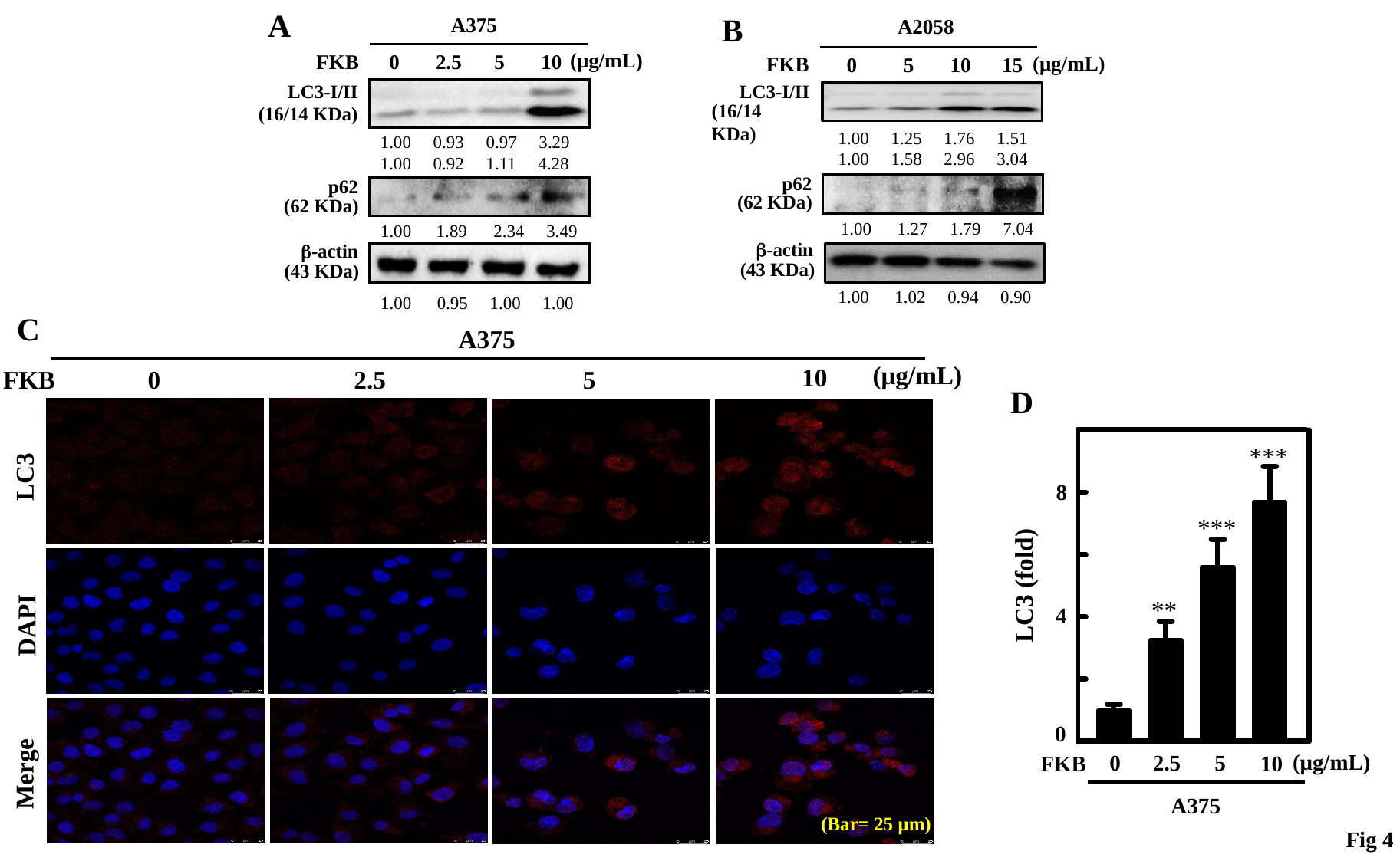

A
A375
 (μg/mL)
 0 2.5 5 10
FKB
LC3-I/II
(16/14 KDa)
1.00 0.93 0.97 3.29
1.00 0.92 1.11 4.28
p62
(62 KDa)
1.00 1.89 2.34 3.49
b-actin
(43 KDa)
1.00 0.95 1.00 1.00
B
A2058
(μg/mL)
 FKB
 0 5 10 15
LC3-I/II
(16/14 KDa)
1.00 1.25 1.76 1.51
1.00 1.58 2.96 3.04
p62
(62 KDa)
1.00 1.27 1.79 7.04
b-actin
(43 KDa)
1.00 1.02 0.94 0.90
C
A375
 (μg/mL)
10
FKB
 0
2.5
5
LC3
DAPI
Merge
(Bar= 25 μm)
D
***
8
***
LC3 (fold)
**
4
0
5
 0
2.5
10
 (μg/mL)
FKB
A375
Fig 4

## Slide 2
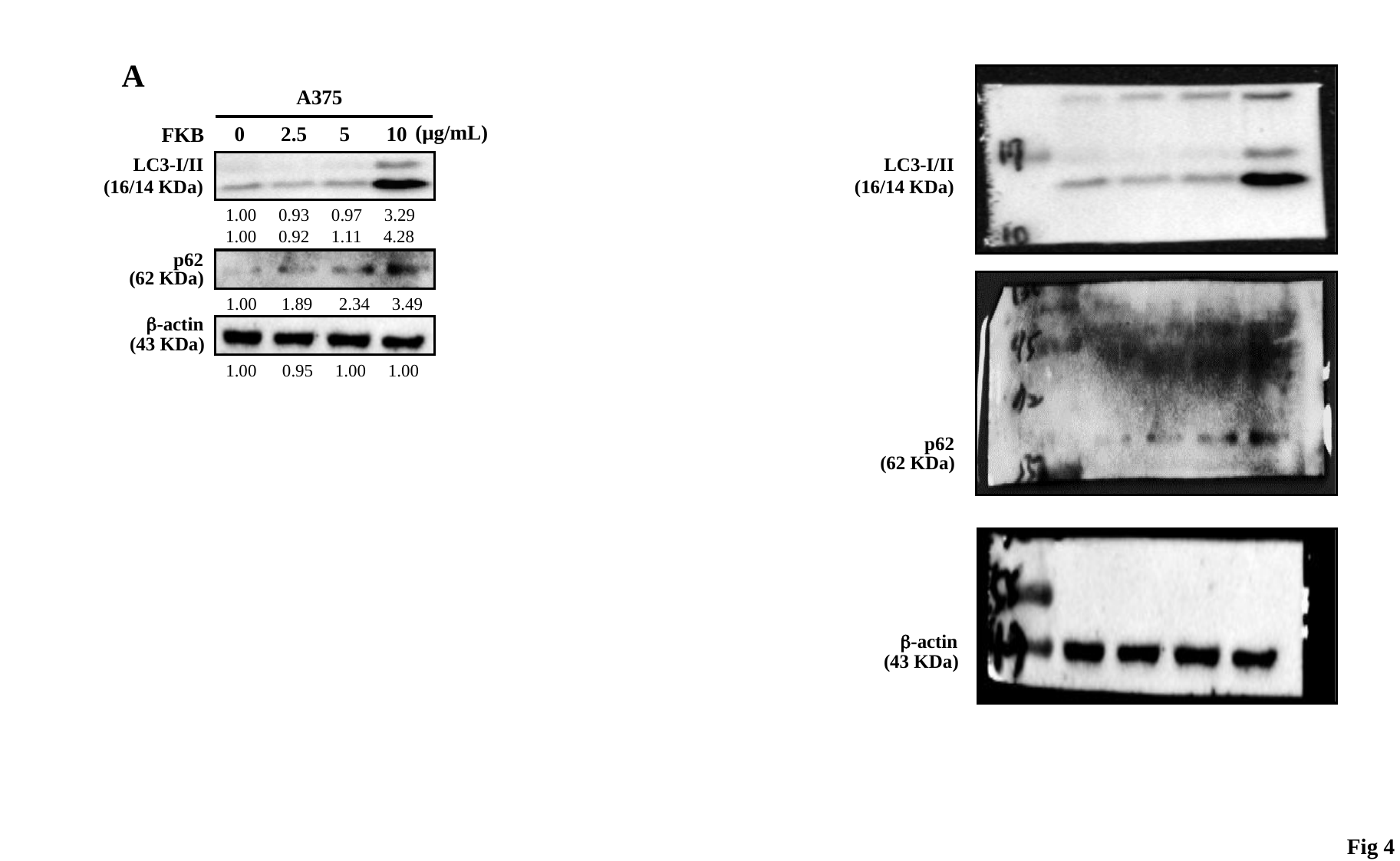

A
A375
 (μg/mL)
 0 2.5 5 10
FKB
LC3-I/II
LC3-I/II
(16/14 KDa)
(16/14 KDa)
1.00 0.93 0.97 3.29
1.00 0.92 1.11 4.28
p62
(62 KDa)
1.00 1.89 2.34 3.49
b-actin
(43 KDa)
1.00 0.95 1.00 1.00
p62
(62 KDa)
b-actin
(43 KDa)
Fig 4

## Slide 3
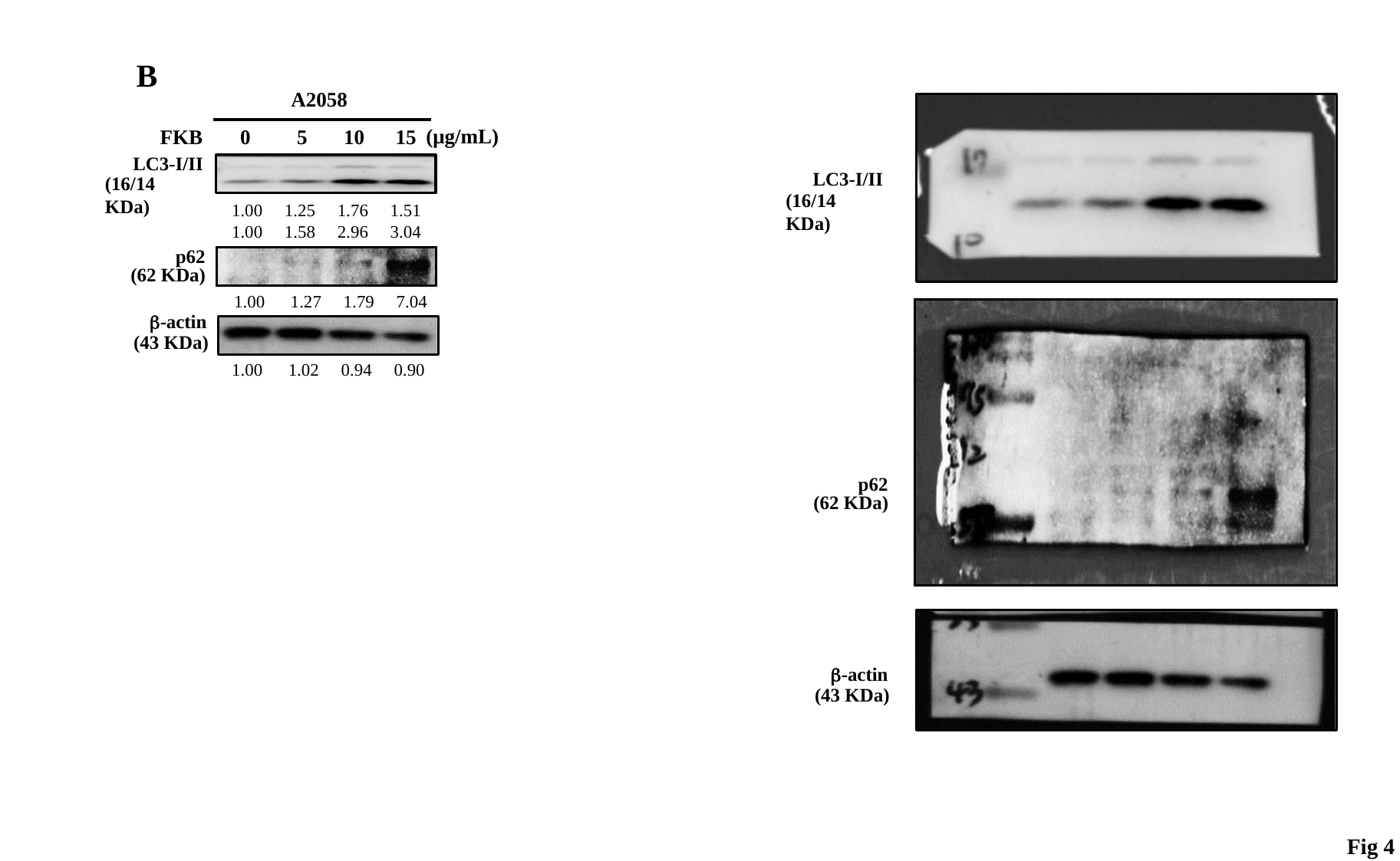

B
A2058
(μg/mL)
 FKB
 0 5 10 15
LC3-I/II
(16/14 KDa)
1.00 1.25 1.76 1.51
1.00 1.58 2.96 3.04
p62
(62 KDa)
1.00 1.27 1.79 7.04
b-actin
(43 KDa)
1.00 1.02 0.94 0.90
LC3-I/II
(16/14 KDa)
p62
(62 KDa)
b-actin
(43 KDa)
Fig 4
