# Supplementary material for: The In Vitro and In Vivo Anticancer Properties of Chalcone Flavokawain B through Induction of ROS-Mediated Apoptotic and Autophagic Cell Death in Human Melanoma Cells
Source: Cancers (Basel). 2020 Oct 12;12(10):2936. doi: 10.3390/cancers12102936 (PMC7600613; doi:10.3390/cancers12102936)
Supplement: Supplementary file 1 [file cancers-12-02936-s001.zip › Fig-S6.pptx]

## Slide 1
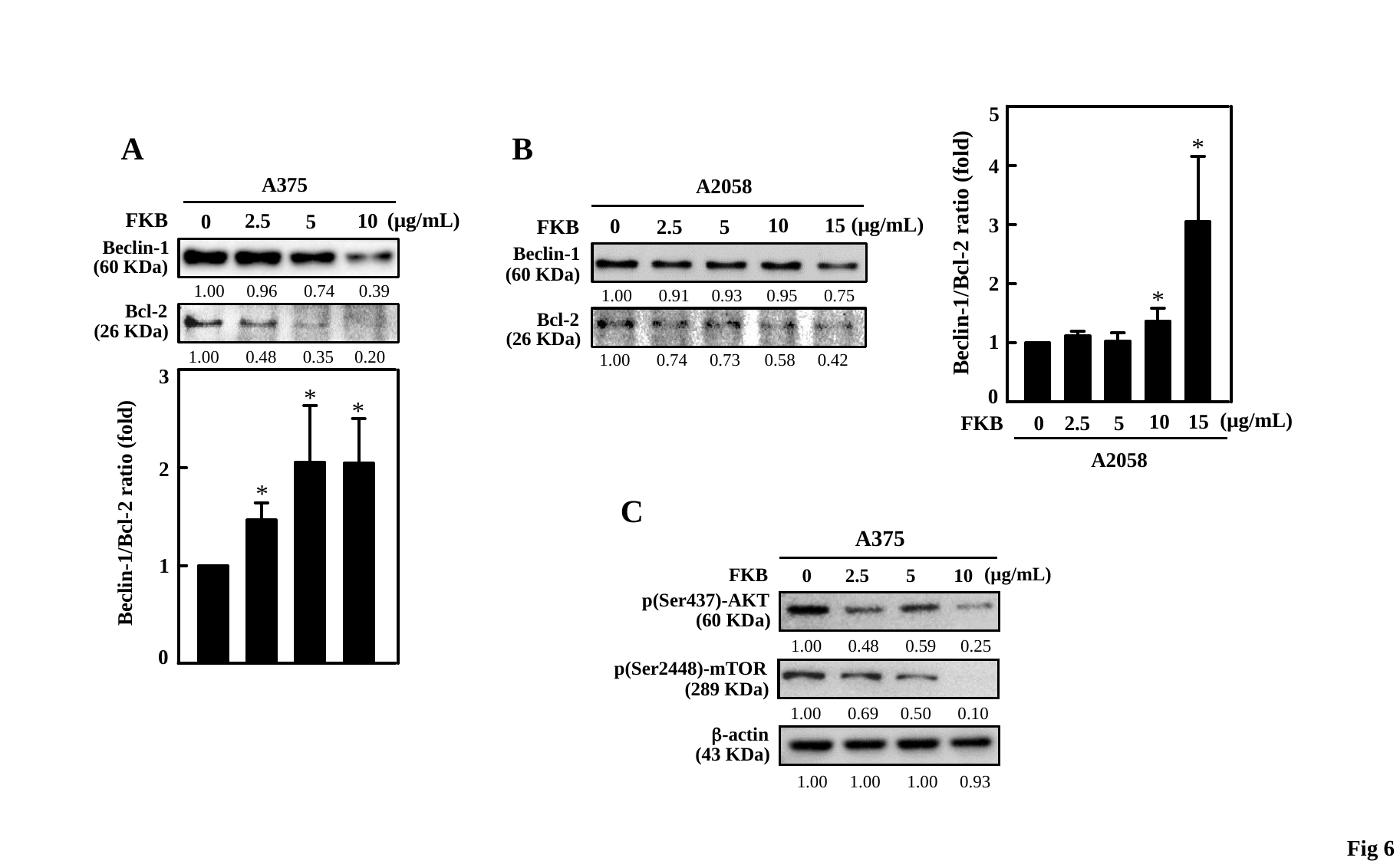

5
*
4
3
Beclin-1/Bcl-2 ratio (fold)
2
*
1
0
10
15
 (μg/mL)
0
5
2.5
FKB
A2058
B
A2058
10
15
0
5
 (μg/mL)
2.5
FKB
Beclin-1
(60 KDa)
1.00 0.91 0.93 0.95 0.75
Bcl-2
(26 KDa)
1.00 0.74 0.73 0.58 0.42
A
A375
2.5
10
5
0
 (μg/mL)
FKB
Beclin-1
(60 KDa)
1.00 0.96 0.74 0.39
Bcl-2
 (26 KDa)
1.00 0.48 0.35 0.20
3
*
*
2
*
Beclin-1/Bcl-2 ratio (fold)
1
0
C
A375
 (μg/mL)
FKB
 0 2.5 5 10
p(Ser437)-AKT
(60 KDa)
1.00 0.48 0.59 0.25
p(Ser2448)-mTOR
(289 KDa)
1.00 0.69 0.50 0.10
b-actin
(43 KDa)
1.00 1.00 1.00 0.93
Fig 6

## Slide 2
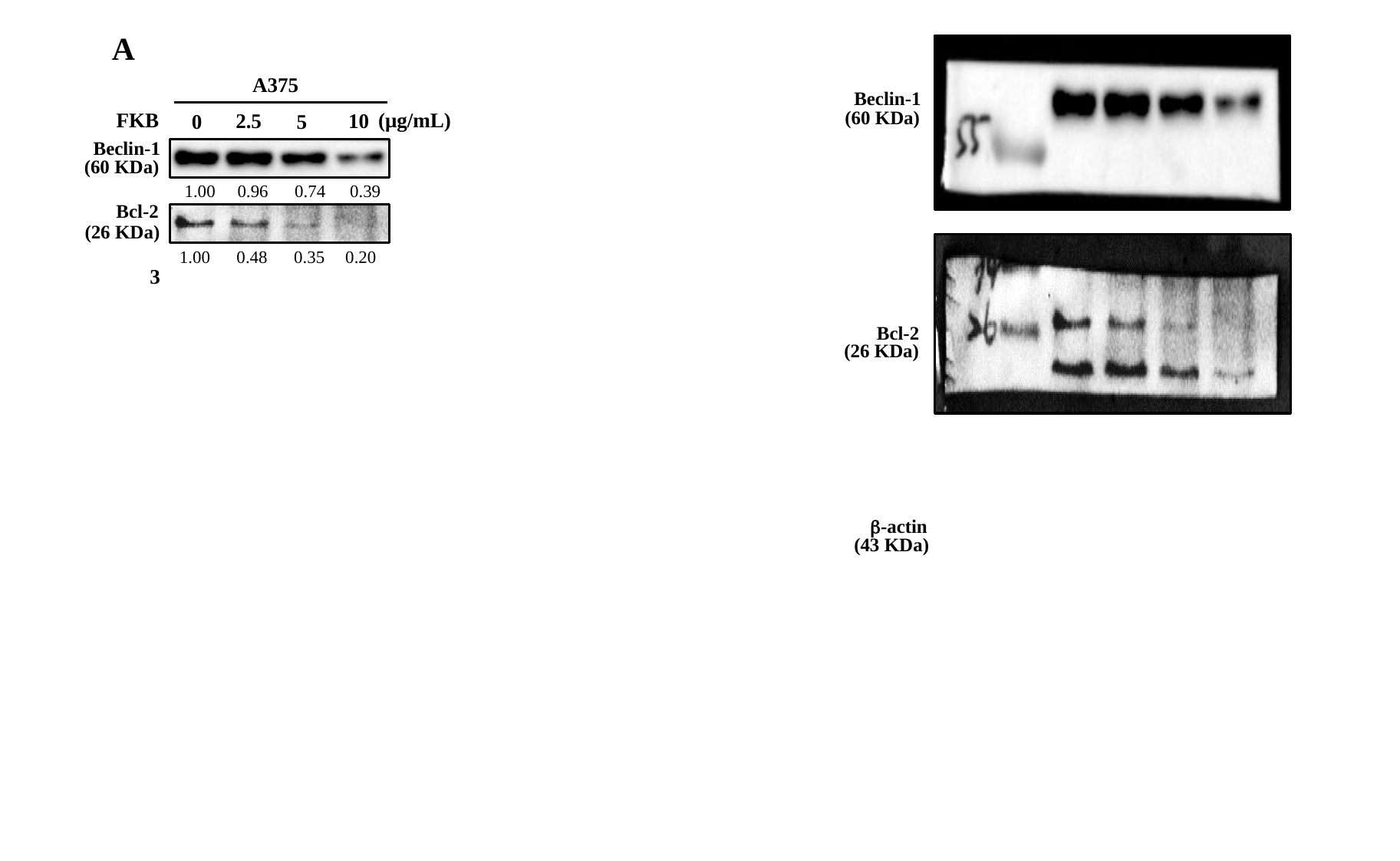

A
A375
Beclin-1
(60 KDa)
2.5
10
5
0
 (μg/mL)
FKB
Beclin-1
(60 KDa)
1.00 0.96 0.74 0.39
Bcl-2
 (26 KDa)
1.00 0.48 0.35 0.20
3
Bcl-2
 (26 KDa)
b-actin
(43 KDa)

## Slide 3
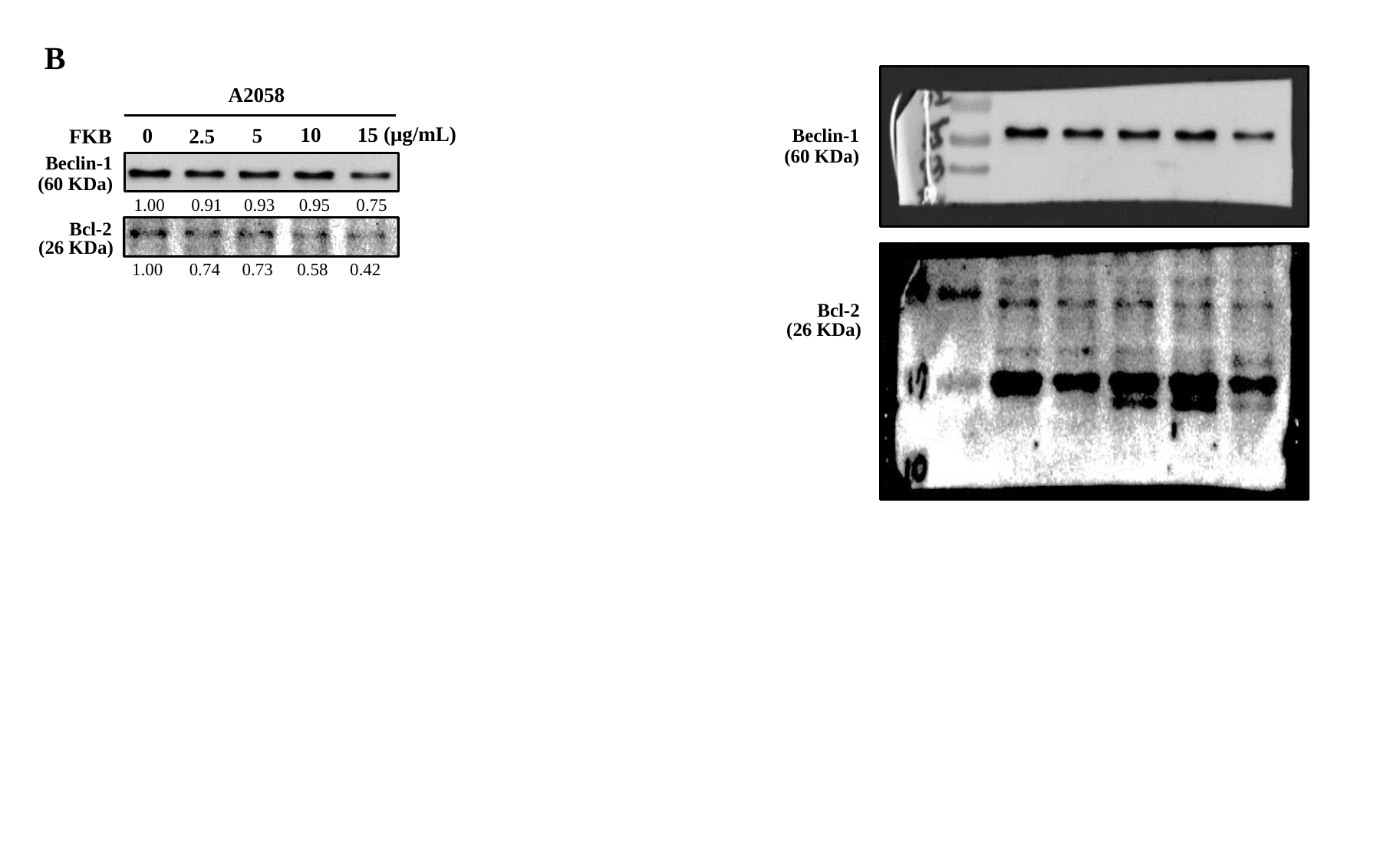

B
A2058
10
15
0
5
 (μg/mL)
2.5
FKB
Beclin-1
(60 KDa)
1.00 0.91 0.93 0.95 0.75
Bcl-2
(26 KDa)
1.00 0.74 0.73 0.58 0.42
Beclin-1
(60 KDa)
Bcl-2
(26 KDa)

## Slide 4
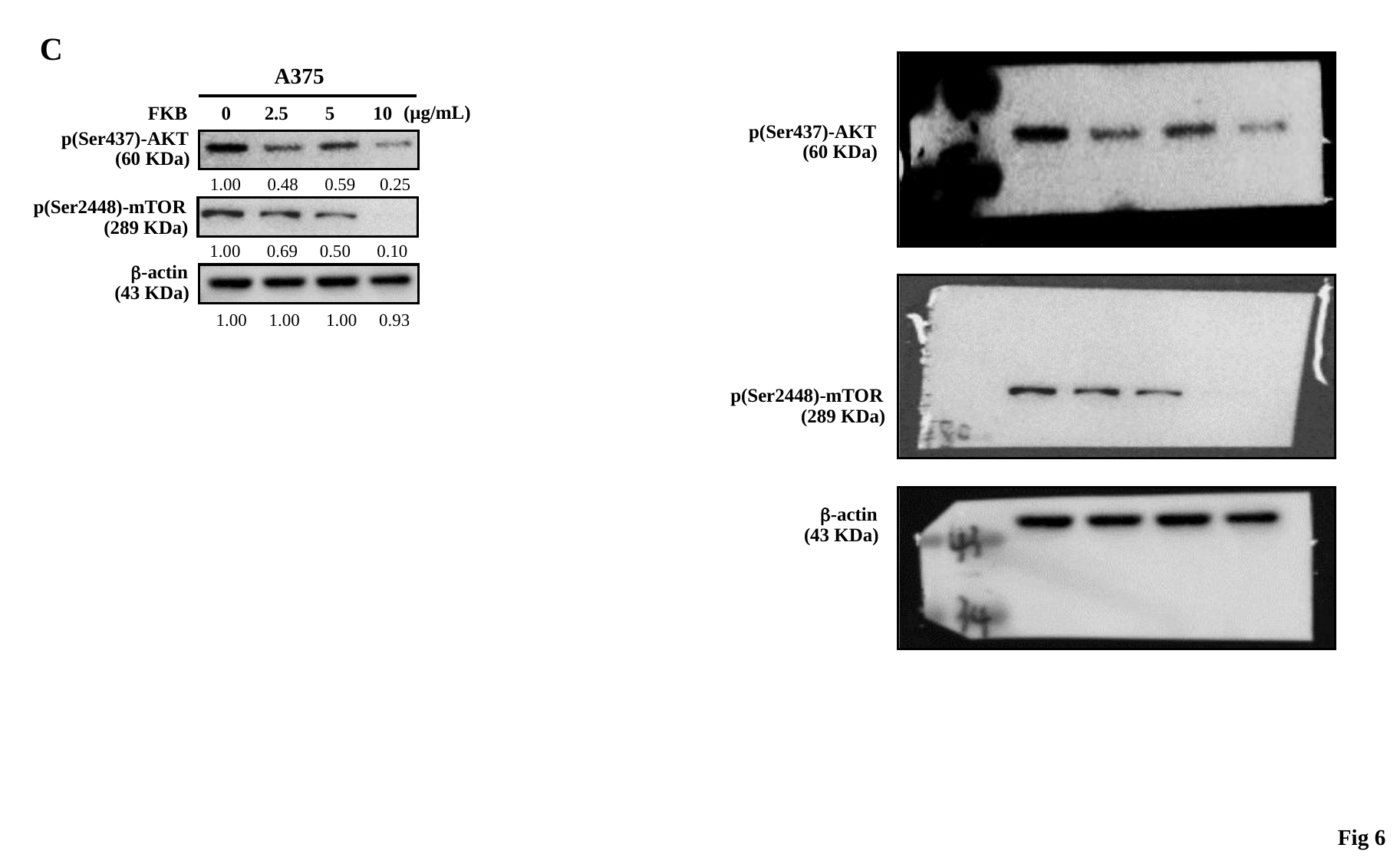

C
A375
 (μg/mL)
FKB
 0 2.5 5 10
p(Ser437)-AKT
(60 KDa)
1.00 0.48 0.59 0.25
p(Ser2448)-mTOR
(289 KDa)
1.00 0.69 0.50 0.10
b-actin
(43 KDa)
1.00 1.00 1.00 0.93
p(Ser437)-AKT
(60 KDa)
p(Ser2448)-mTOR
(289 KDa)
b-actin
(43 KDa)
Fig 6
