# Supplementary material for: The In Vitro and In Vivo Anticancer Properties of Chalcone Flavokawain B through Induction of ROS-Mediated Apoptotic and Autophagic Cell Death in Human Melanoma Cells
Source: Cancers (Basel). 2020 Oct 12;12(10):2936. doi: 10.3390/cancers12102936 (PMC7600613; doi:10.3390/cancers12102936)
Supplement: Supplementary file 1 [file cancers-12-02936-s001.zip › Fig-S10.pptx]

## Slide 1
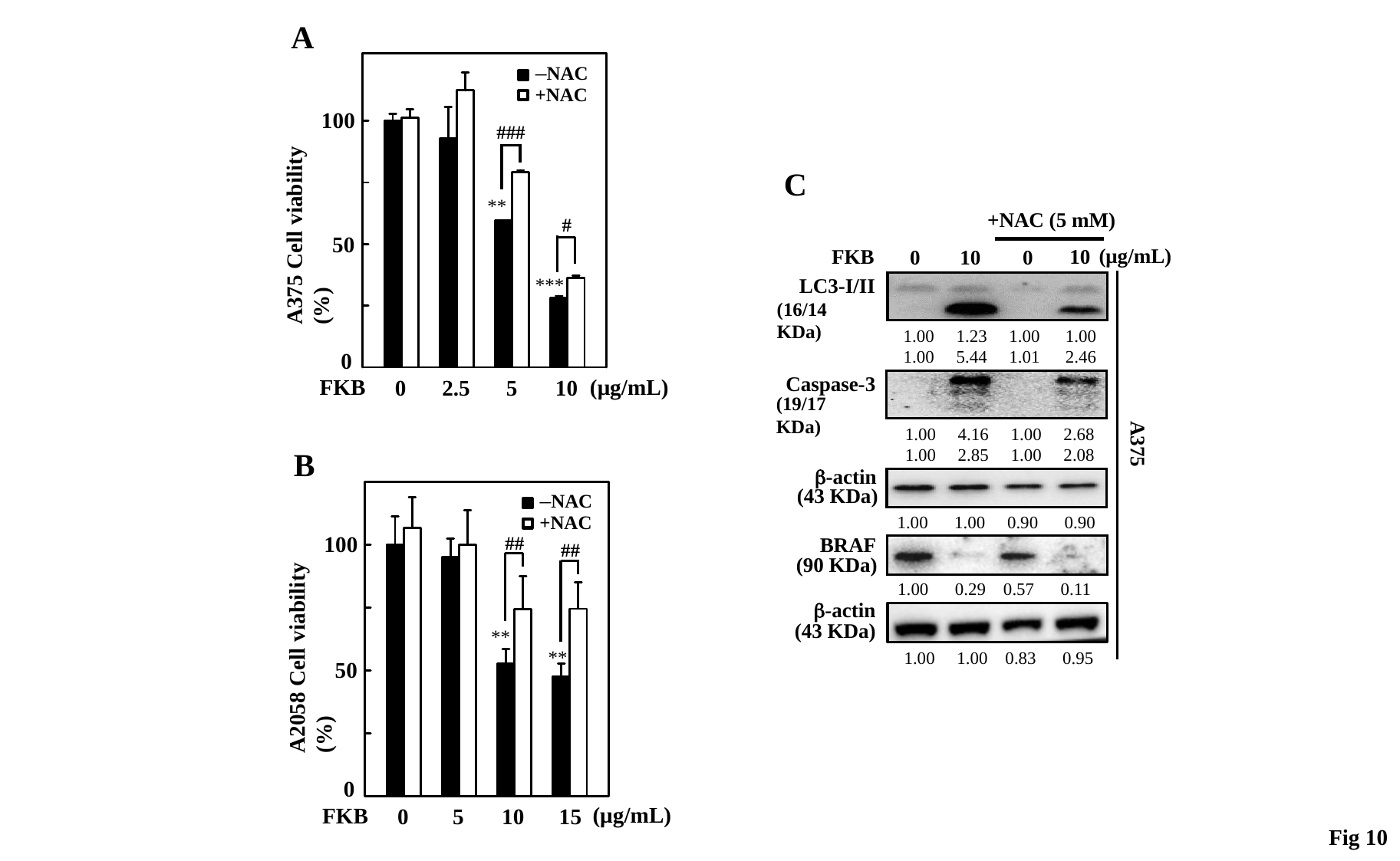

A
─NAC
+NAC
A375 Cell viability (%)
100
###
**
#
50
***
0
(μg/mL)
FKB
0
 2.5
 5
10
C
+NAC (5 mM)
(μg/mL)
10
FKB
0
0
10
LC3-I/II
(16/14 KDa)
1.00 1.23 1.00 1.00
1.00 5.44 1.01 2.46
Caspase-3
(19/17 KDa)
1.00 4.16 1.00 2.68
1.00 2.85 1.00 2.08
A375
b-actin
(43 KDa)
1.00 1.00 0.90 0.90
BRAF
(90 KDa)
1.00 0.29 0.57 0.11
b-actin
(43 KDa)
1.00 1.00 0.83 0.95
B
─NAC
+NAC
A2058 Cell viability (%)
100
##
##
**
**
50
0
(μg/mL)
FKB
0
 5
 10
15
Fig 10
| 0 | 2.5 | 10 | 20 | ZVAD | ZVAD+2.5 | ZVAD+5 | ZVAD+10 |
| --- | --- | --- | --- | --- | --- | --- | --- |
| 100 | 85.2532 | 50.13912 | 34.11241 | 101.6138 | 69.67168 | 55.31441 | 44.2404 |

## Slide 2
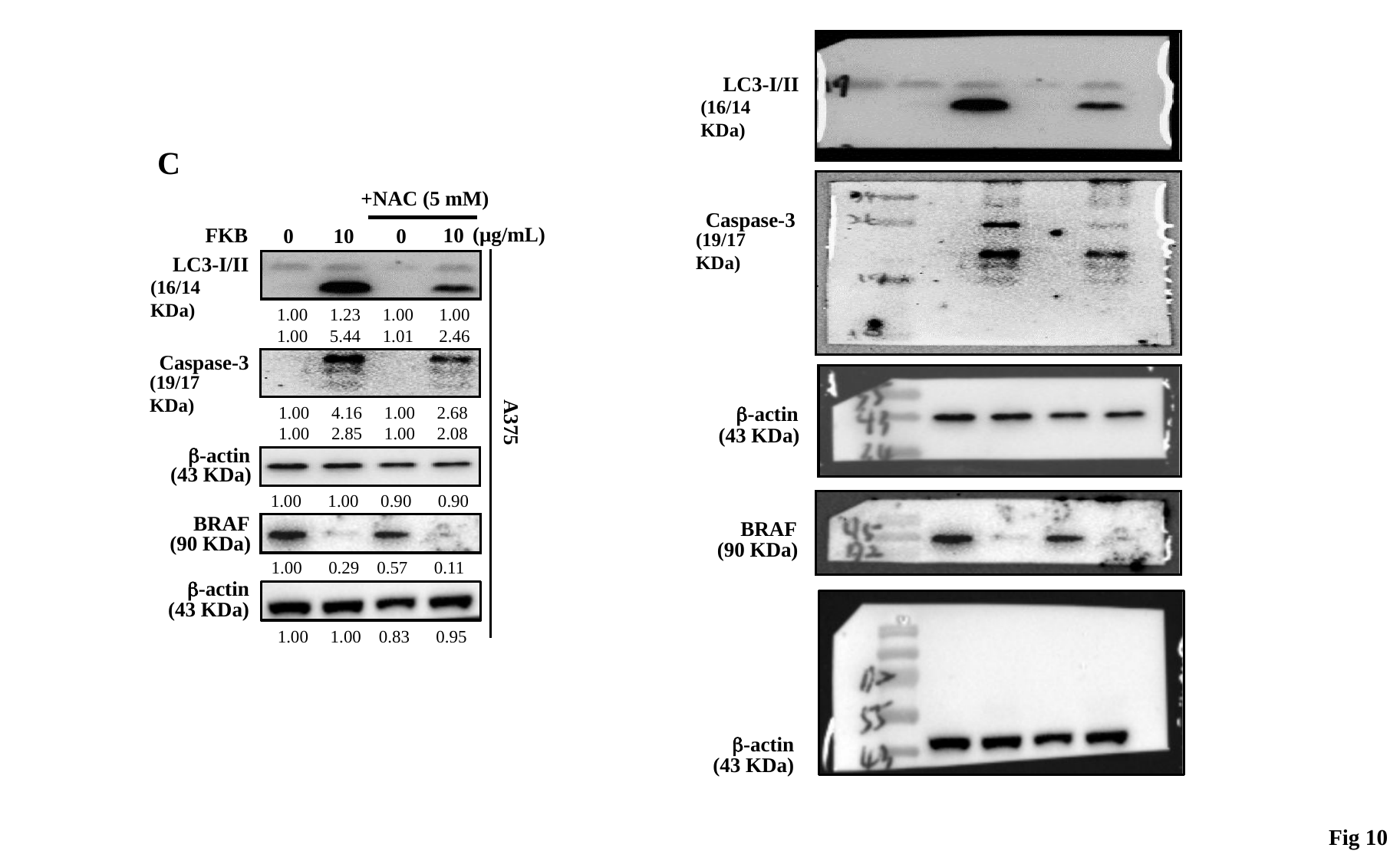

LC3-I/II
(16/14 KDa)
C
+NAC (5 mM)
(μg/mL)
10
FKB
0
0
10
LC3-I/II
(16/14 KDa)
1.00 1.23 1.00 1.00
1.00 5.44 1.01 2.46
Caspase-3
(19/17 KDa)
1.00 4.16 1.00 2.68
1.00 2.85 1.00 2.08
A375
b-actin
(43 KDa)
1.00 1.00 0.90 0.90
BRAF
(90 KDa)
1.00 0.29 0.57 0.11
b-actin
(43 KDa)
1.00 1.00 0.83 0.95
Caspase-3
(19/17 KDa)
b-actin
(43 KDa)
BRAF
(90 KDa)
b-actin
(43 KDa)
Fig 10
| 0 | 2.5 | 10 | 20 | ZVAD | ZVAD+2.5 | ZVAD+5 | ZVAD+10 |
| --- | --- | --- | --- | --- | --- | --- | --- |
| 100 | 85.2532 | 50.13912 | 34.11241 | 101.6138 | 69.67168 | 55.31441 | 44.2404 |
