# Supplementary material for: The In Vitro and In Vivo Anticancer Properties of Chalcone Flavokawain B through Induction of ROS-Mediated Apoptotic and Autophagic Cell Death in Human Melanoma Cells
Source: Cancers (Basel). 2020 Oct 12;12(10):2936. doi: 10.3390/cancers12102936 (PMC7600613; doi:10.3390/cancers12102936)
Supplement: Supplementary file 1 [file cancers-12-02936-s001.zip › Fig-S11.pptx]

## Slide 1
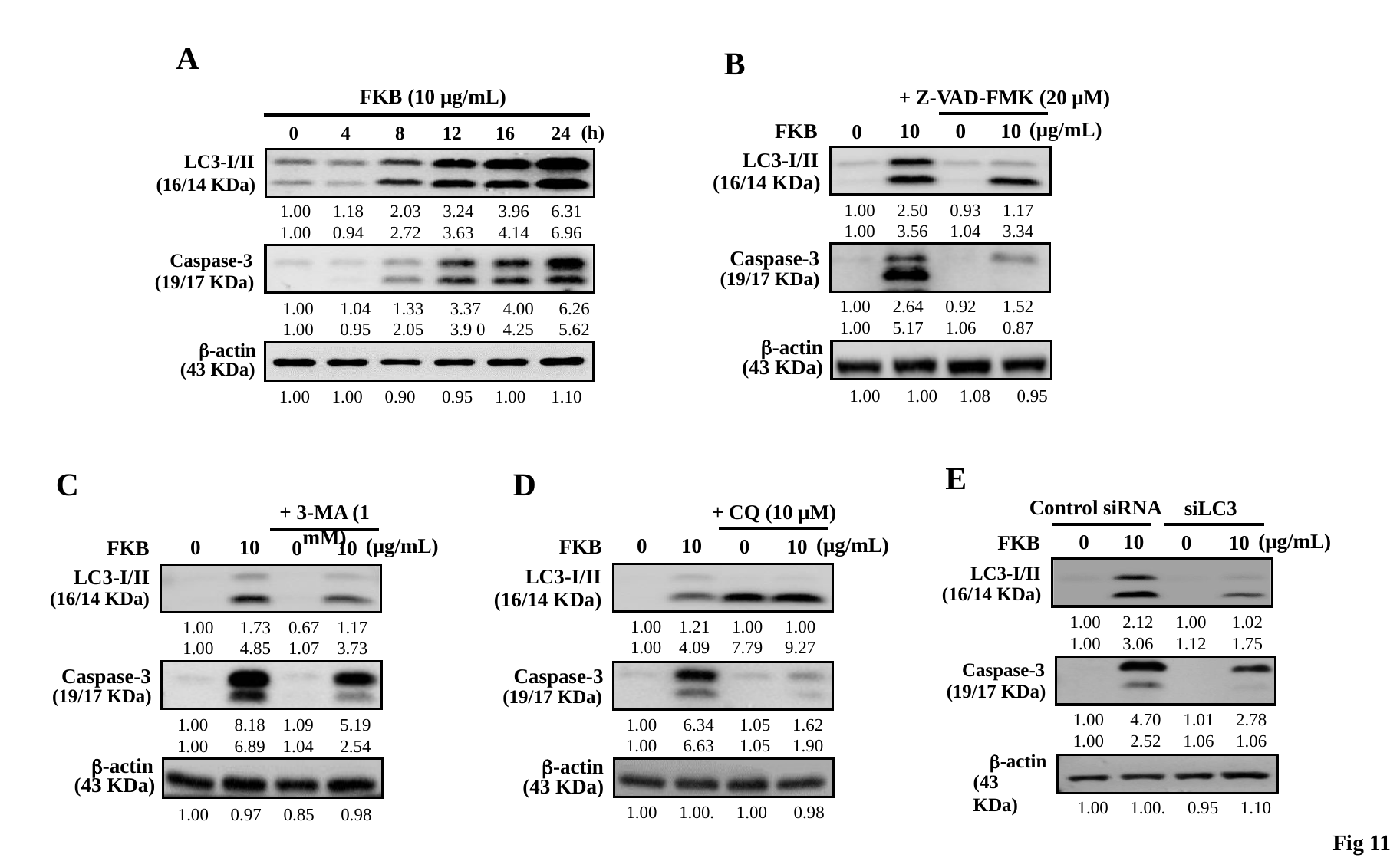

A
FKB (10 μg/mL)
 (h)
0
4
8
12
16
24
LC3-I/II
(16/14 KDa)
1.00 1.18 2.03 3.24 3.96 6.31
1.00 0.94 2.72 3.63 4.14 6.96
Caspase-3
(19/17 KDa)
1.00 1.04 1.33 3.37 4.00 6.26
1.00 0.95 2.05 3.9 0 4.25 5.62
b-actin
(43 KDa)
1.00 1.00 0.90 0.95 1.00 1.10
B
+ Z-VAD-FMK (20 μM)
(μg/mL)
0
10
10
FKB
0
LC3-I/II
(16/14 KDa)
1.00 2.50 0.93 1.17
1.00 3.56 1.04 3.34
Caspase-3
(19/17 KDa)
1.00 2.64 0.92 1.52
1.00 5.17 1.06 0.87
b-actin
(43 KDa)
1.00 1.00 1.08 0.95
E
Control siRNA
siLC3
(μg/mL)
0
10
FKB
0
10
LC3-I/II
(16/14 KDa)
1.00 2.12 1.00 1.02
1.00 3.06 1.12 1.75
Caspase-3
(19/17 KDa)
1.00 4.70 1.01 2.78
1.00 2.52 1.06 1.06
b-actin
(43 KDa)
1.00 1.00. 0.95 1.10
D
+ CQ (10 μM)
(μg/mL)
0
10
FKB
0
10
LC3-I/II
(16/14 KDa)
1.00 1.21 1.00 1.00
1.00 4.09 7.79 9.27
Caspase-3
(19/17 KDa)
1.00 6.34 1.05 1.62
1.00 6.63 1.05 1.90
b-actin
(43 KDa)
1.00 1.00. 1.00 0.98
C
+ 3-MA (1 mM)
(μg/mL)
0
10
10
FKB
0
LC3-I/II
(16/14 KDa)
1.00 1.73 0.67 1.17
1.00 4.85 1.07 3.73
Caspase-3
(19/17 KDa)
1.00 8.18 1.09 5.19
1.00 6.89 1.04 2.54
b-actin
(43 KDa)
1.00 0.97 0.85 0.98
Fig 11

## Slide 2
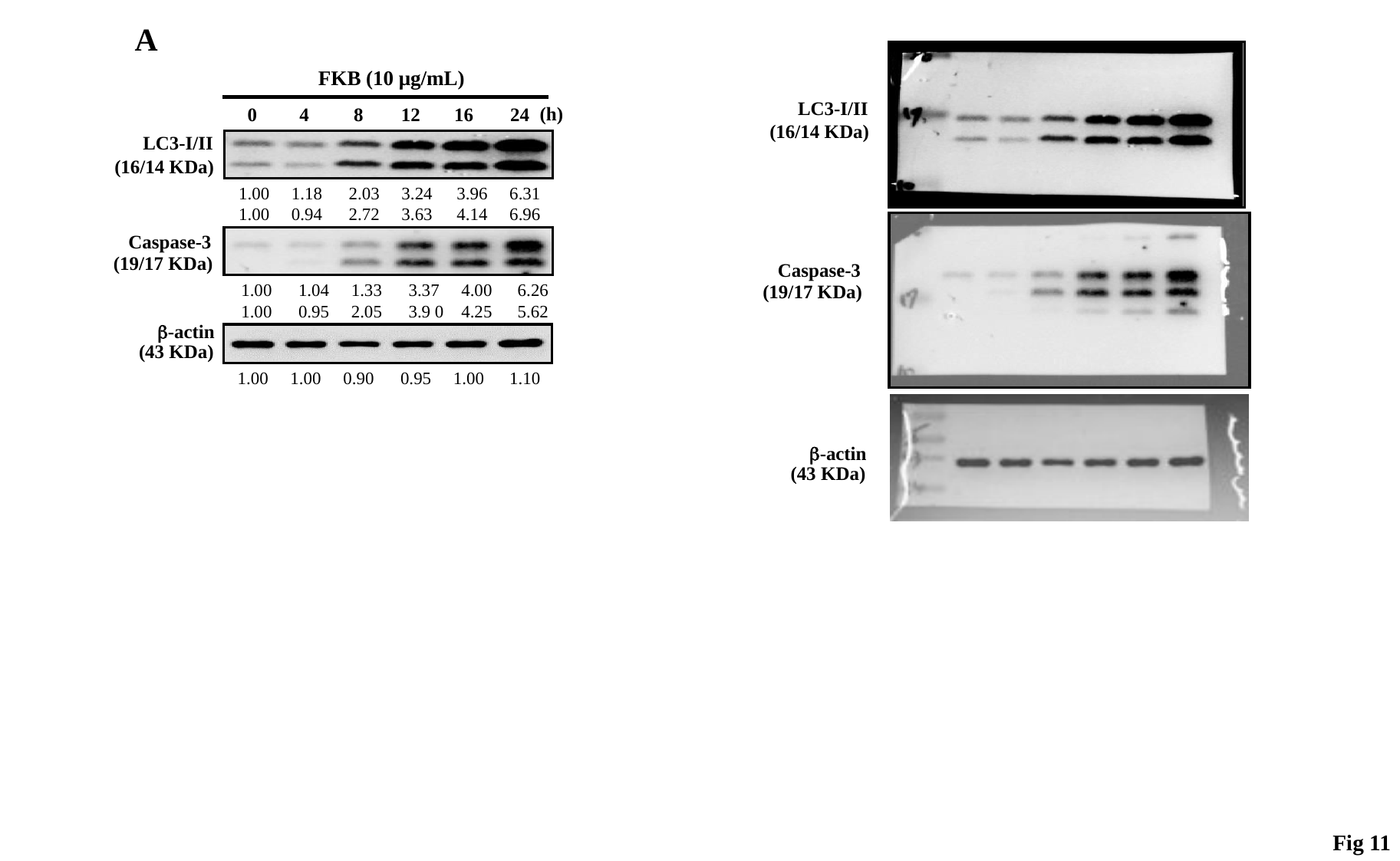

A
FKB (10 μg/mL)
 (h)
0
4
8
12
16
24
LC3-I/II
(16/14 KDa)
1.00 1.18 2.03 3.24 3.96 6.31
1.00 0.94 2.72 3.63 4.14 6.96
Caspase-3
(19/17 KDa)
1.00 1.04 1.33 3.37 4.00 6.26
1.00 0.95 2.05 3.9 0 4.25 5.62
b-actin
(43 KDa)
1.00 1.00 0.90 0.95 1.00 1.10
LC3-I/II
(16/14 KDa)
Caspase-3
(19/17 KDa)
b-actin
(43 KDa)
Fig 11

## Slide 3
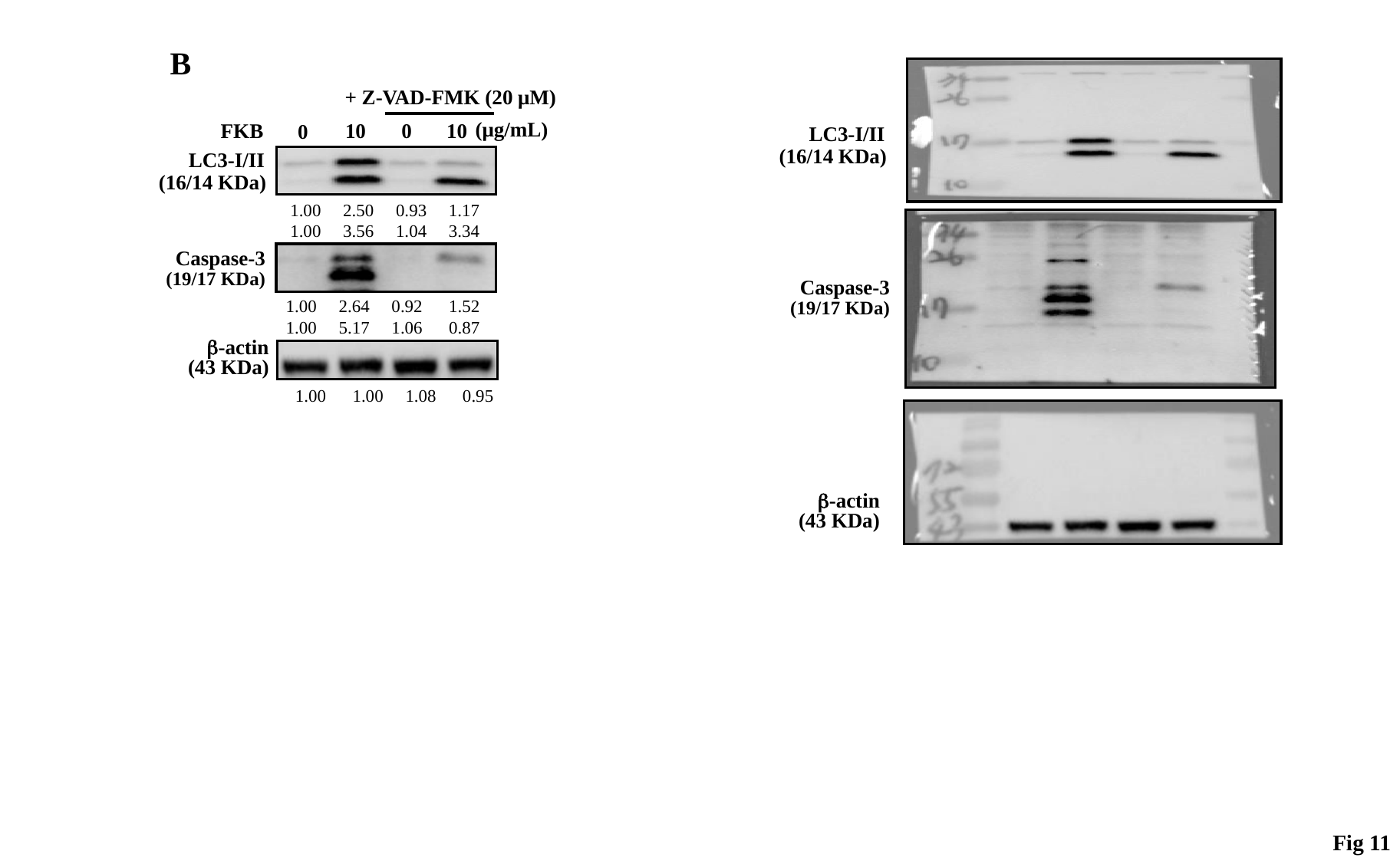

B
+ Z-VAD-FMK (20 μM)
(μg/mL)
0
10
10
FKB
0
LC3-I/II
(16/14 KDa)
1.00 2.50 0.93 1.17
1.00 3.56 1.04 3.34
Caspase-3
(19/17 KDa)
1.00 2.64 0.92 1.52
1.00 5.17 1.06 0.87
b-actin
(43 KDa)
1.00 1.00 1.08 0.95
LC3-I/II
(16/14 KDa)
Caspase-3
(19/17 KDa)
b-actin
(43 KDa)
Fig 11

## Slide 4
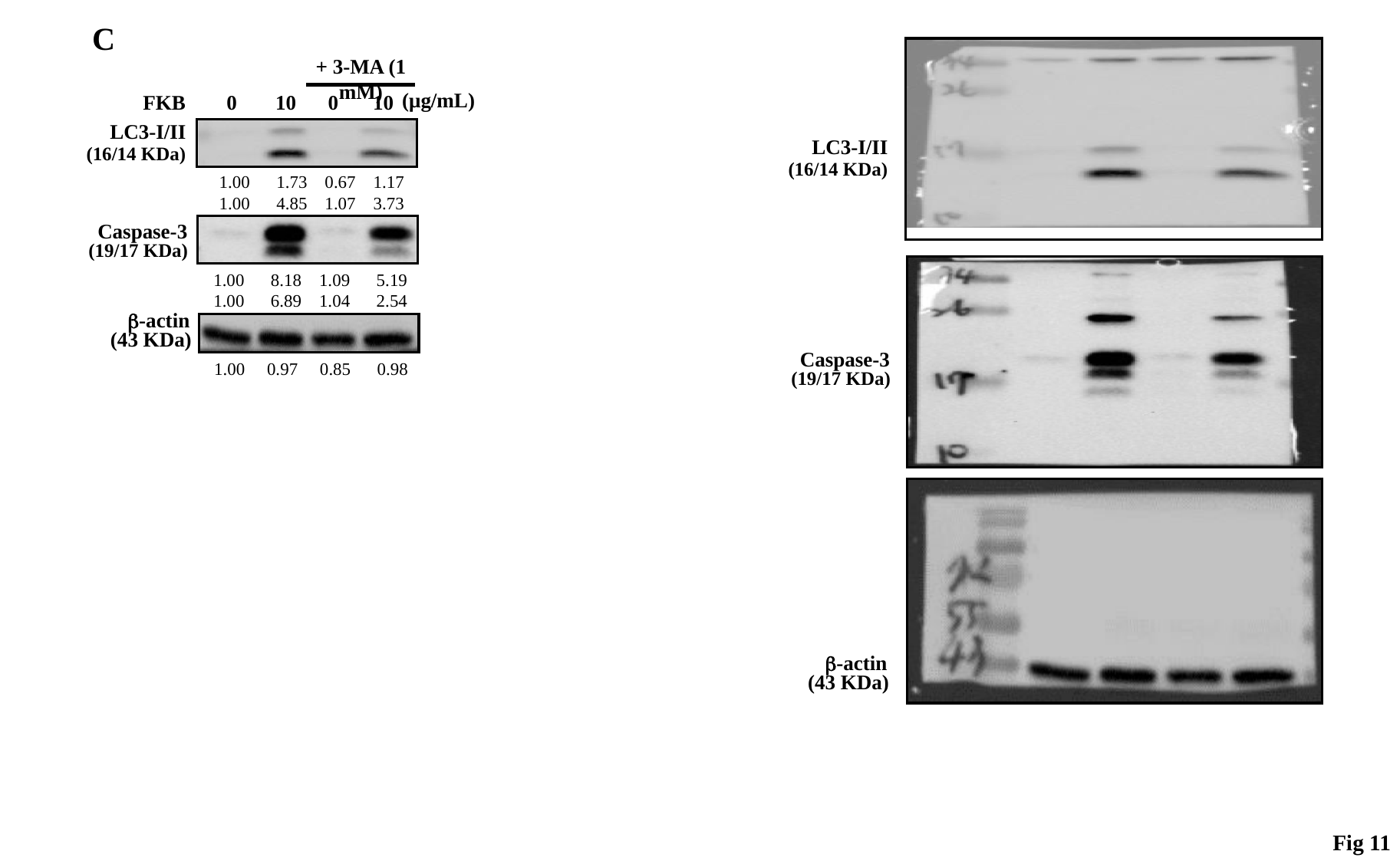

C
+ 3-MA (1 mM)
(μg/mL)
0
10
10
FKB
0
LC3-I/II
(16/14 KDa)
1.00 1.73 0.67 1.17
1.00 4.85 1.07 3.73
Caspase-3
(19/17 KDa)
1.00 8.18 1.09 5.19
1.00 6.89 1.04 2.54
b-actin
(43 KDa)
1.00 0.97 0.85 0.98
LC3-I/II
(16/14 KDa)
Caspase-3
(19/17 KDa)
b-actin
(43 KDa)
Fig 11

## Slide 5
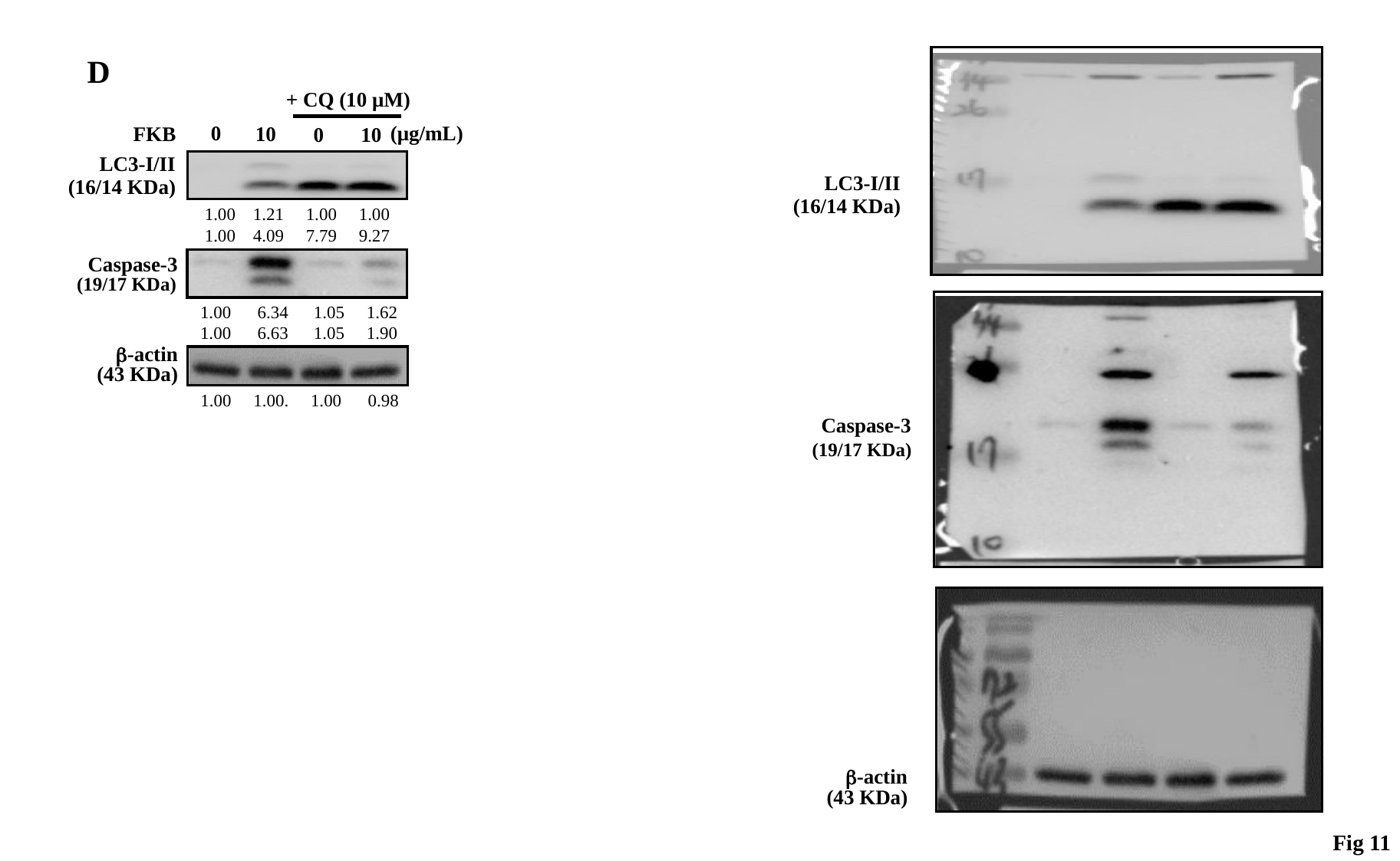

D
+ CQ (10 μM)
(μg/mL)
0
10
FKB
0
10
LC3-I/II
(16/14 KDa)
1.00 1.21 1.00 1.00
1.00 4.09 7.79 9.27
Caspase-3
(19/17 KDa)
1.00 6.34 1.05 1.62
1.00 6.63 1.05 1.90
b-actin
(43 KDa)
1.00 1.00. 1.00 0.98
LC3-I/II
(16/14 KDa)
Caspase-3
(19/17 KDa)
b-actin
(43 KDa)
Fig 11

## Slide 6
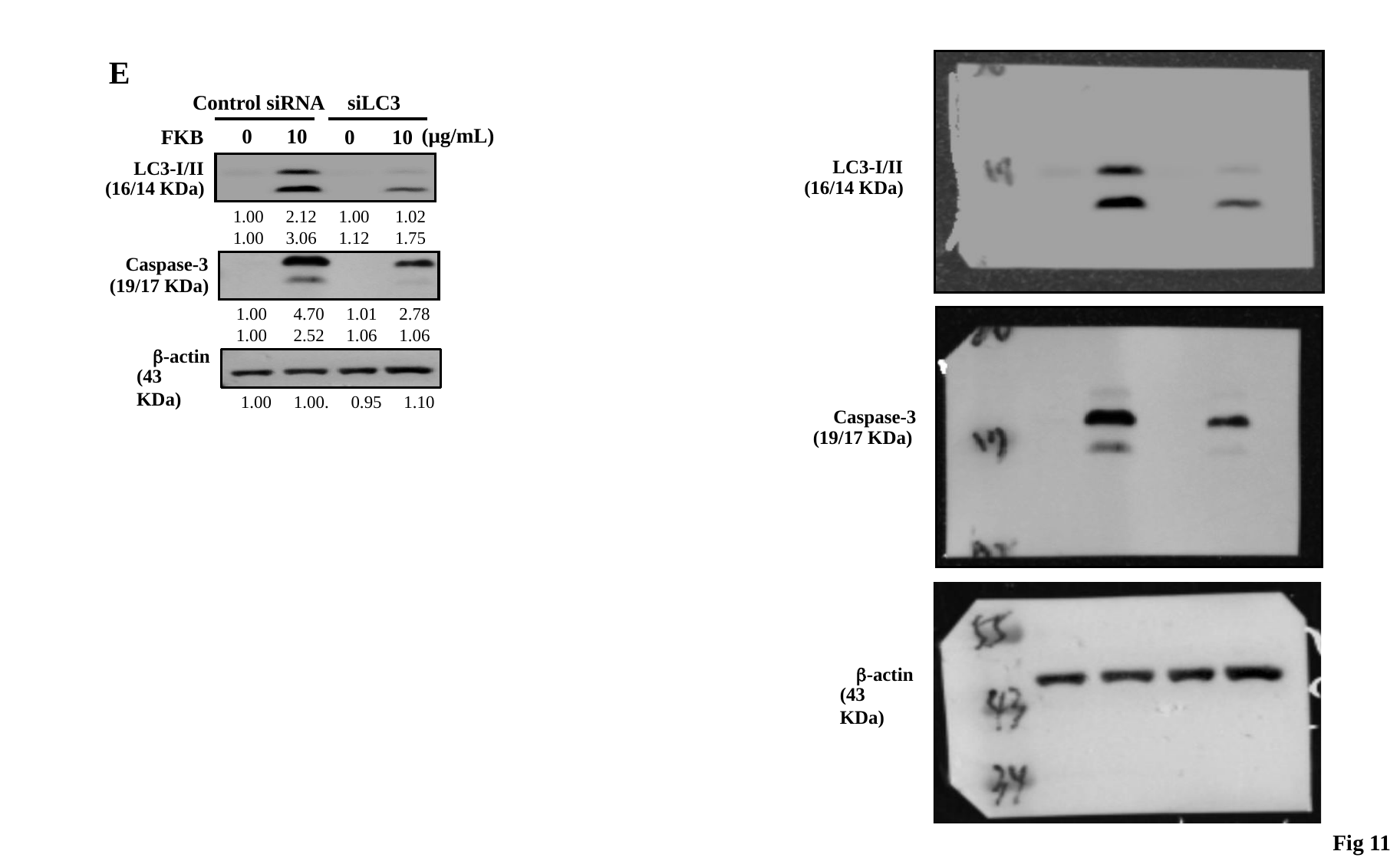

E
Control siRNA
siLC3
(μg/mL)
0
10
FKB
0
10
LC3-I/II
(16/14 KDa)
1.00 2.12 1.00 1.02
1.00 3.06 1.12 1.75
Caspase-3
(19/17 KDa)
1.00 4.70 1.01 2.78
1.00 2.52 1.06 1.06
b-actin
(43 KDa)
1.00 1.00. 0.95 1.10
LC3-I/II
(16/14 KDa)
Caspase-3
(19/17 KDa)
b-actin
(43 KDa)
Fig 11
